# Privacy in targeted advertising on mobile devices: a survey

**DOI:** 10.1007/s10207-022-00655-x

**Published:** 2022-12-24

**Authors:** Imdad Ullah, Roksana Boreli, Salil S. Kanhere

**Affiliations:** 1 College of Computer Engineering and Sciences, Prince Sattam bin Abdulaziz University, 11942 Al-Kharj, Saudi Arabia; 2 TMPP Pty. Ltd., Sydney, Australia; 3 The University of New South Wales (UNSW), Sydney, Australia

**Keywords:** Targeted advertising, Privacy, Information leakage, Privacy threats, Tracking, Crypto billing

## Abstract

Targeted advertising has transformed the marketing landscape for a wide variety of businesses, by creating new opportunities for advertisers to reach prospective customers by delivering personalised ads, using an infrastructure of a number of intermediary entities and technologies. The advertising and analytics companies collect, aggregate, process, and trade a vast amount of users’ personal data, which has prompted serious privacy concerns among both individuals and organisations. This article presents a comprehensive survey of the privacy risks and proposed solutions for targeted advertising in a mobile environment. We outline details of the information flow between the advertising platform and ad/analytics networks, the profiling process, the measurement analysis of targeted advertising based on user’s interests and profiling context, and the ads delivery process, for both in-app and in-browser targeted ads; we also include an overview of data sharing and tracking technologies. We discuss challenges in preserving the mobile user’s privacy that include threats related to private information extraction and exchange among various advertising entities, privacy threats from third-party tracking, re-identification of private information and associated privacy risks. Subsequently, we present various techniques for preserving user privacy and a comprehensive analysis of the proposals based on such techniques; we compare the proposals based on the underlying architectures, privacy mechanisms, and deployment scenarios. Finally, we discuss the potential research challenges and open research issues.

## Introduction

Online advertising has become a prevalent marketing tool, commanding the majority of spending and taking over from the traditional broadcast advertising in newspapers, television and radio. According to Statista^1^, in 2022, 62% of global ad spending is forecast to be on internet ads, while television will have around 23%. This is primarily due to the ability of online ad platforms to tailor or personalise ads, and thereby target specific customer segments. Targeted advertising is based on Big data analytics, where user’s personal information is collected and processed to enable segmenting users into groups based on interests, location, or personal attributes like age, gender, etc., with a varying size of the selected customer segment, down to the level of an individual.

The most significant platform from which personal data are collected and subsequently used for targeted ads is a mobile device, including mobile phones or tablets, due to its widespread and almost continuous use by a huge audience of potential ad recipients. A recent report [1] lists that 69% of users’ digital media time is actually spent on mobile phones only and consequently recommends tailoring targeted ads for mobile devices. Although mobile users are still utilising browsers to access various online sites, applications (*apps*) are increasingly replacing the generic browser functionality. Currently, millions of mobile *apps* can be downloaded via various *app* marketplaces like the Google Play Store and the Apple App Store. Specifically, in 2021, there were around 230 billion mobile *app* downloads [2].

Most mobile *apps* contain at least one ad library (including analytics^2^ libraries) [3] that enables targeted (or behavioural) mobile advertising to a wide range of audiences. The information about users and their online behaviour is collected through the ad library API calls [4]. This includes information inference based on monitoring ads displayed during browsing sessions [5, 6]. The Advertising and Analytics (A &A) companies like Google Analytics and Flurry using this framework are working to increase their revenue by providing ad libraries that the *apps* developers use to serve ads. In the process of data monetisation, the advertising/analytics companies aggressively look for all possible ways to gather personal data from users [7], including purchasing users’ personal data from third parties.

The collection and use of personal data pose serious threats to the privacy of users [8–13], when websites or *apps* indicating sensitive information are used as the basis for profiling, e.g. a gaming *app* showing a gambling problem. Privacy concerns have been increasingly recognised by policy-makers, with the introduction of anti-tracking laws, gradually making the use of some third-party tracking techniques used for interest-based targeting obsolete. E.g. Google has announced Chrome’s ‘Cookie Apocalypse’, planning to phase out support for third-party cookies by 2022^3^. Subsequently, instead of relying on third-party data, the A &A companies are increasingly using first-party data and shifting towards maintaining their own Data Management Platforms (DMPs) and Demand-Side Platforms (DSPs)^4^ to brand their data and measure performance in a ‘cookie-less’ world. In a stronger push towards increased user’s privacy control over the collection and use of their data, Apple^5^ has recently introduced the Identification for Advertisers (IDFA) opt-in overhaul in iOS 14.5, which will have a significant impact on targeted ads and mobile ad/data attribution. This has created a very public feud with one of the largest social networks (and private data collection companies), Facebook [14], highlighting two different business approaches in regards to privacy and user targeting.

Privacy is also a subject of legal frameworks in a large number of countries, e.g. the ‘EU General Data Protection Regulation (GDPR)’ [15], ‘The Privacy Act in Australia’ [16]. In the US, state laws regulate general privacy protection, e.g. the ‘California Consumer Privacy Act (CCPA)’ [17].

Overall, regardless of the technological and policy changes, protecting users’ personal data while having effective targeting is important to both the advertising networks and mobile users. Mobile users do want to view relevant (interest-based) ads, provided that their information is not exposed to the outside world including the advertising companies. Advertising networks can only be effective if they deliver the most relevant ads to users, to achieve better view/click-through rates, while protecting the interactions between mobile users, advertisers, and publishers/ad networks.

In this paper, we survey the threats and solutions related to privacy in mobile targeted advertising. We first present a survey of the existing literature on privacy risks, resulting from the information flow between the A &A companies, temporal tracking of users regarding both their activities, and the outcomes of targeting them with personalised ads. We then describe, for both *in-app* (note that we interchangeably use ‘mobile’ and ‘*in-app*’) and *in-browser* targeted ads: the user profiling process, data collection, and tracking mechanism, the ad delivery process and the process of ad characterisation. We outline the privacy threats posed by the A &A companies as a result of targeting; in particular (to prove the privacy leakage), we demonstrate, using experimental evaluation, how private information is extracted and exchanged among various entities in an advertising system including third-party tracking and highlight the associated privacy risks. Subsequently, we provide an overview of privacy-preserving techniques applicable to online advertising, including differential privacy, anonymisation, proxy-based solutions, k-anonymity, i.e. generalisation and suppression, obfuscation, and crypto-based techniques such as Private Information Retrieval (PIR) and Blockchain-based techniques. We also survey the proposed privacy-preserving advertising systems and provide a comparative analysis of the proposals, based on the underlying architectures, the privacy techniques used, and the deployment scenarios. Finally, we discuss the research challenges and open research issues.

Prior survey works focused on more generic privacy topics, e.g. [7, 18–20]. To the best of our knowledge, this paper is the first comprehensive review of privacy techniques and solutions in mobile targeted advertising.

This article is organised as follows. Section 2 presents a comprehensive methodology for conducting this survey. In Sect. 3, we introduce the mobile advertising ecosystem, its operation for the ad delivery process, profiling process and characterisation of *in-app* and *in-browser* ads. Section 4 discusses the technical and comprehensive understanding of ad network operations for targeted ads. Section 5 presents privacy threats and information leakage in online advertising systems. Section 6 presents a comprehensive comparative analysis of various privacy-preserving advertising systems. Various open research issues are outlined in Sect. 7. We conclude in Sect. 8.

## Methodology

In this section, we outline the methodology used to select the prior research work and other references included in our paper.

We note that this survey focuses on data minimisation privacy-enhancing technologies, rather than the other privacy protection goals as defined in policy frameworks [15]. We consider representative works for both surveys of privacy in related fields to mobile advertising and in privacy technologies and systems applicable to our focus area.

Previous literature surveys related to our work consider broader areas of Personalization and privacy [18], Online advertising [7], the privacy-personalisation trade-offs [19] and Online behavioural advertising [20]. Our focus is on specific issues and technologies related to mobile (in-app) advertising.

Our starting points are previous works, as per [21]. Our literature search included queries performed on Google Scholar, the databases provided by IEEE Xplore, Elsevier, Springer, ScienceDirect, and ProQuest. In addition, we searched for relevant articles in Google Scholar in order to find articles published with other publishers, e.g. MDPI. We used the following combination of keywords: ‘Private/Secure Targeted/Mobile/Online behavioural advertising’, and ‘Targeted/Mobile/Online behavioural advertising’ along with ‘Private information retrieval, Privacy, Information leakage, Privacy threats, Tracking, Billing, Cryptocurrency, Blockchain, RTB, Characterisation, Obfuscation, Differential privacy’.

The initial search was based on database queries performed on the databases provided by Inspec^6^ and DBLP^7^, as well as the publication databases of the two publishers ACM^8^ and IEEE^9^. These databases were chosen because of the high quality of the publications available in or referenced by them.

More specifically, we consider the following conditions to select and include articles in our survey: (1) The published work must be within the domain of advertising systems, (2) the ad system may be browser-based or app-based, (3) the research article may have addressed part of a private/secure advertising system, e.g. private profiling, (4) the research article may be related to performance measurements, advertising measurements or traffic analysis, etc., (5) we also consider supporting articles to elaborate a particular concept or theory, and (6) we include conference papers, journals, books, early access articles, magazines, and survey articles only.

We note that this paper is a ‘traditional’ survey, rather than a Systematic literature review and as such has the associated limitations [22].

In the following section, we introduce the mobile advertising ecosystem, its operation for the ad delivery process, the profiling process, and characterisation of targeted ads.

## The mobile advertising network

The ad network ecosystem involves different entities which comprise the advertisers, ad agencies and brokers, ad networks delivering ads, *analytics* companies, publishers, and the end customers to whom ads are delivered [23]. For the case of large publishers, the ads may be served both by the publishers and the advertisers [24]; consequently, the ad ecosystem includes a number of interactions between different parties.

### The advertising ecosystem

**Fig. 1:**
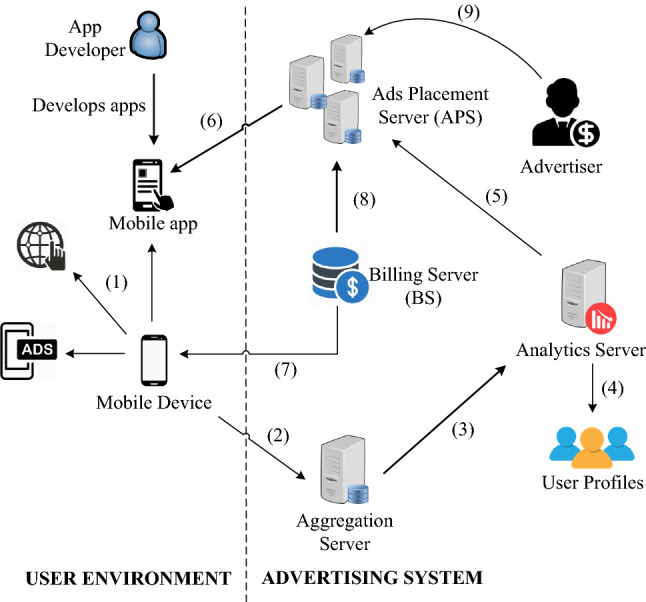
The mobile advertising ecosystem, including the information flow among different parties. (1) Data collection and tracking, (2) Send tracking data to Aggregation server, (3) Forward usage info to Analytics server, (4) User profiling, (5) Send profiling info to APS, (6) Deliver targeted/generic ads, (7) Billing for *apps* developer, (8) Billing for Ad System, (9) Advertiser who wishes to advertise with Ad system

A typical mobile ad ecosystem (both for *in-app* and *in-browser* ads) and the information flow among different parties is presented in Fig. 1. A user has a number of *apps* installed on their mobile device, that are utilised with specific frequency. As demonstrated in [25], most mobile *apps* include *analytics* Software Development Kit (SDK) and as such both report their activity and send ad requests to the *analytics* and ad network. This network comprises the Aggregation server, *analytics* server, Billing server, and the Ads Placement Server (APS). Collected data, that relates to usage of mobile *apps* and the success of displayed ads, is used by the ads *analytics* server to develop user profiles (associated with specific mobile devices and corresponding users). A user profile comprises a number of *interests*, that indicates the use of related *apps*, e.g. sports, business, etc., constructed by e.g. Google Advertising network for Mobile (AdMob)^10^ and Flurry [26] (note that the latter is only visible to *app* developers). *Targeted* ads are served to mobile users according to their individual profiles. We note that other, i.e. *generic* ads are also delivered [27]. The Billing server includes the functionality related to monetising *Ad impressions* (i.e. ads displayed to the user in specific *apps*) and *Ad clicks* (user action on selected ads); further discussion over ads *billing* is given in Sect. 3.5.

### User profiling

Advertising systems rely on user *profiling* and *tracking* to tailor ads to users with specific interests and to increase their advertising revenue. Following, we present the user *profiling* process, in particular, how the user profile is *established*, various criteria, and how it *evolves* over time.

#### Profile establishment

**Fig. 2:**
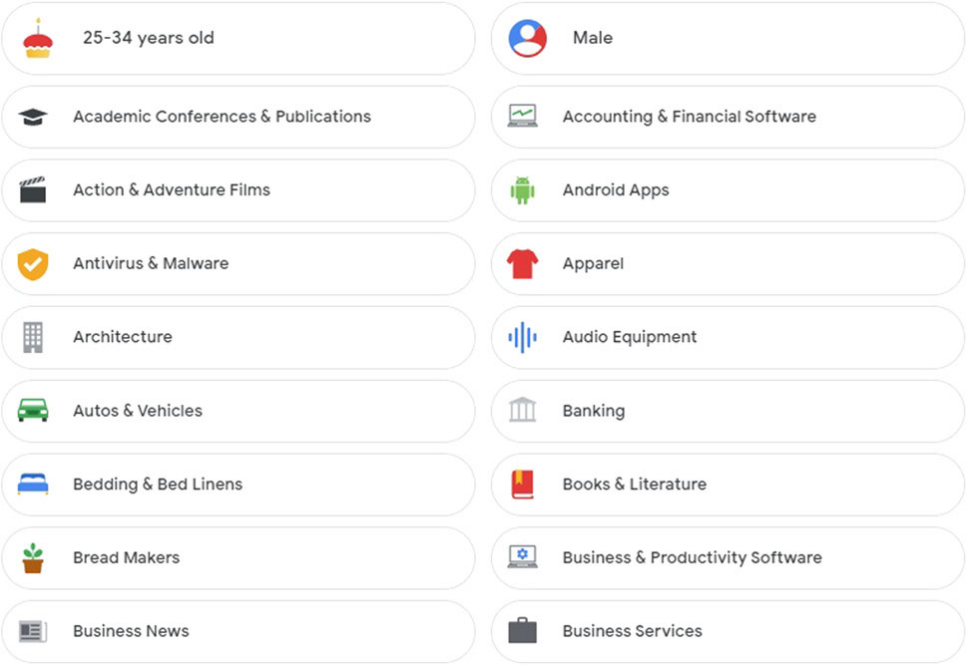
An (anonymous) example of a user profile estimated by Google as a results of *Web & App* activity

The advertising companies, e.g. Google, profile users based on the information they add to their Google account, data collected from other advertisers that partner with Google, and its estimation of user’s interests based on mobile *apps* and websites that agree to show Google ads. An example profile estimated by Google with various demographics (e.g. gender, age-ranks) and profiling interests (e.g. Autos & Vehicles) is shown in Fig. 2. It is assumed that there is a *mapping* of the *Apps profile* $K_a$ (the *apps* installed on a user mobile device) to an *Interests profile* $I_g$ (such an example set of interests is shown in Fig. 2) defined by advertising (e.g. Google) and *analytics* companies, i.e. $K_a \rightarrow I_g$. This information is used by the *analytics* companies to individually characterise users’ interests across the advertising ecosystem.

This *mapping* includes the conversion of the *apps* categories $\varPhi _j$ (where $j=1,\ldots , \tau$ and $\tau$ is the set of different categories in a marketplace) to interest categories $\varPsi _l$ ($l=1,\ldots , \epsilon$. $\epsilon$ is the set of interest categories that are defined by the *analytics* company). This *mapping* converts an *app* ${a_{i,j}} \in {S_a}$ to interests set $S_g^{i,j}$ after a specific level of activity ${t_{est}}$. The ${t_{est}}$ is the *establishment threshold*, i.e. time an *app* should be used in order to establish profile’s interests. The result of this *mapping* is a set of interests, called *Interests profile* $I_g$. Google profile interests^11^ are grouped, hierarchically, under various interest categories, with specific interests.

Furthermore, the ads *targeting* is based on demographics i.e. age range, gender, etc., to reach a specific set of potential customers. Google^12^ presents a comprehensive set of *demographic targeting* options for search campaigns, ads display, etc. The demographics *D* are typically grouped in diverse categories, with specific options, such as age-ranges, e.g. ‘18–24’, ‘25–34’, ‘35–44’, ‘45–54’, ‘55–64’, ‘65 or more’, and gender e.g. ‘Male’, ‘Female’, besides other options, e.g. location, household income, etc. Recall that the user *profiling* results when the user device interacts with Google *analytics* via AdMob SDK [9] for various activities. The ‘My Google Activity’^13^ shows a complete set of ‘*Web & App* activities’ that helps Google make services more useful, such as helping rediscover the things already searched for, read, and watched.

**Fig. 3:**
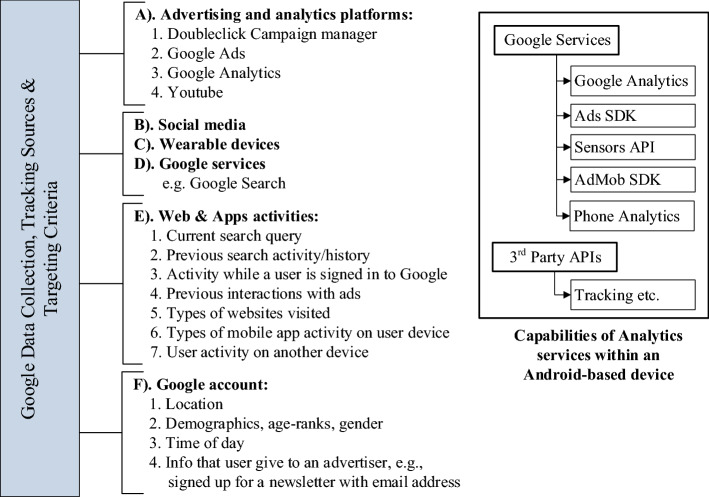
Google’s data collection and *tracking* sources for *targeting* users with personalised ads (left) and tracking capabilities of analytics libraries enabled within mobile devices (right)

The various platforms in Google’s ad system are shown in Fig. 3; these are used for collecting profiling data and *targeting* users with personalised ads. Data collection is enabled via various tools, e.g. the Android/iOS SDKs helps aggregate the ‘*Web & Apps* activities’ and users’ interactions with the *analytics* servers, cookies, *conversion tracking*^14^, web histories and searches, user’s interactions with received ads, etc. Likewise, Google’s connected home devices and services^15^ collect data using cameras, microphones and other sensors to provide personalised features and services^16^. Google Takeout^17^ can be used to export a copy of content (up to several GBs of data) to the user’s Google Account for backup or third-party services. Furthermore, this includes the data from a wide-range of Google products personalised for specific users, such as email conversations (including ‘Trash’ and ‘Spam’ folder emails), calendar, browsing history, location traces and photos.

#### Profile evolution

An updates to a user profile is effected each time the users’ behaviour is varied, e.g. when the *apps* context changes, resulting in non-existing interests in the current profile. Let $S_a$ be the existing set of apps that had produced a $I_g$ interest profile. Let the user start using a new set of *apps* $S{'_a}$ that has no overlap with $S_a$, i.e. $S'_a \subset {{{\mathscr {A}}}}\setminus {S_a}$ where ${{\mathscr {A}}}$ is the set of *apps* in an *app* market. The set $S{'_a}$ is converted to non-existing interests after a certain level of activity, i.e. the ${t_{evo}}$ *evolution threshold*, which is the time required to evolve new profile’s interests $I{'_g}$. After the profile evolution process, the final *Interests profile* $I_g^f$ combines the older interests derived during the profile *establishment* $I_g$ and the *evolution* $I{'_g}$ processes.

#### Profile development process

**Fig. 4:**
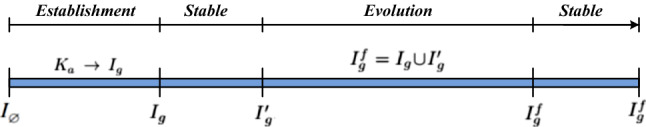
Profiling processes, i.e. profile *establishment* & *evolution*. The ${I_\emptyset }$ indicates the initial empty profile before using any *apps*. The *Interest profiles* $I_g$ or $I_g^f$ stays the same during the *stable* states where additional activities of the same *apps* do not have any effect on the user profiles

There is a minimum level of activity of the installed *apps* in an *Apps profile* required to *establish* an *Interests profile*. Recall that, to generate interests, the *apps* need to have the AdMob SDK. To verify this, we used 10 test phones and run an overall 1200 *apps* for a duration of 8 days that were selected from 12 *apps* categories. We note that, among the 1200 *apps*, the 1143 *apps* resulted from the *Interest profiles* on all test phones indicating ‘Unknown’ interests. We further note that these *apps* deterministically derive *Interests profile*, i.e. a particular *app* always derives identical interests during the profile *establishment/evolution* process. In addition, we note that the activity level of *apps* must be within a minimum of 24 hours period with a minimum of 24/*n* hours of activity of *n* *apps*, from our experimentations. Using our extensive experimentations, we note that Google Analytics requires this much time in order to determine one’s profiling interests. Hence, for a sophisticated *profiling* and to reflect one’s interests, a user might want to install and use an extensive set of *apps* that would represent one’s interests. Following the 24 hours, the user profile goes in the *stable* where the further activity of the same *apps* has no effect on the profile. Figure 4 shows the *establishment*, *evolution*, and the *stable* states of an *Apps profile* mapped to an *Interests profile*.

During the profile *evolution* process, the *Interests profile* changes by adding new interests when *apps* other than the existing set of *apps* $S_a$ are being utilised. However, we observed these changes in the *Interests profile* taking place in the following 72 h of profile period, rather than the 24 h period of profile *evolution* process; this is when the aggregated profile, i.e. $I_g^f$, becomes *Stable*. Furthermore, running these *apps* on 4th day in order to verify the stability of the resultant aggregated profile, we observed no further changes, as shown in Fig. 4.

### Targeted advertising

Mobile targeted advertising is a crucial factor in increasing revenue (a prediction shows the mobile ad market to grow to $408.58 billion in 2026 [28]) in a mobile *app* ecosystem that provides free services to the smartphone users. This is mainly due to users spending significantly more time on mobile *apps* than on the traditional web. Hence, it is important to deliver ads based on user’s interests (note that targeted advertising is not only used for mobile ads, it is also utilised for in-browser ads). The characterisation of *targeted* advertising, on the user’s side, includes the analysis of the ad-delivery process, to determine what information the mobile *apps* send to the ad network and how effectively they utilise this information for ads *targeting*. Furthermore, the characterisation of mobile targeted ads enables the ad networks to analyse and subsequently enhance the ad delivery process, resulting in improved ad view and click rates.

### Ads selection algorithms

The accurate measurement of the *targeted* advertising is directly related to the ad selection algorithm. Some of the ad selection algorithms perform ad selection based on the user data pattern [29] and the program event analysis [30]; however, the *contextual* and *targeted* advertising is treated differently as they are related to the psyche of the users. Consequently, it has been observed that the activity of users and their demographics strongly influences the ad selection, along with the user clicks of an ad [31, 32]. As an example, a young female that is frequently browsing websites or using mobile *apps* related to the category of *entertainment*, would be more interested in receiving ads related to *entertainment* such as movies, musical instruments, etc.; consequently, it increases the *click-through rates*. Another work [33] builds a game-theoretic model for ad systems competing through *targeted* advertising and shows how it affects the consumers’ search behaviour and purchasing decisions when there are multiple firms in the market. We note that the researchers utilise different ad selection and *targeting* algorithms based on machine learning and data mining techniques.

### Ad billing

Billing is an important part of business models devised by any advertising system that is based on billing their customers for fine-grained use of ad systems and their resources. Specifically, advertisers include various payment settings and payment methods for monetising *ad impressions* and *clicks*.

## Operations of the advertising system

In this section, we discuss the technical aspects of the advertising systems, i.e. the ad delivery process, ads traffic extraction and characterisation, to assist in understanding privacy issues in *targeted* advertising.

### Ad delivery process

**Fig. 5:**
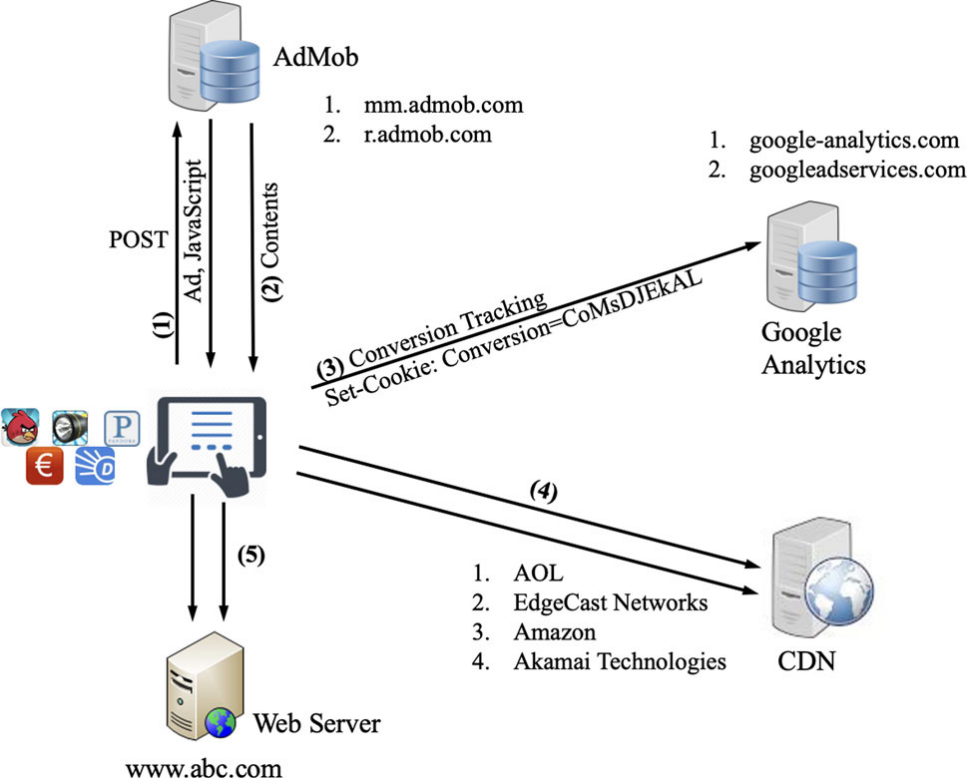
AdMob Ad presentation workflow

We identify the workflow of a mobile *app* requesting a Google AdMob ad and the triggered actions resulting from e.g. a user click (we note that other advertising networks, such as Flurry, use different approaches/messages to request ads and to report ad clicks). Figure 5 describes some of the domains used by AdMob (Google ad servers and AdMob are shown separately for clarity, although both are acquired by Google). As shown in Step 2, an ad is downloaded after the POST method is sent by the mobile phone containing phone version, model, *app* running on a phone, etc. In Step 3, the ad contains the landing page (the web address of an ad-URL) and JavaScript code that is executed where some of the static objects are downloaded (such as a PNG). Following in Step 4, two actions are performed after clicking an ad: a *Conversion* cookie^18^ is set inside the phone and the associated web server is contacted. in addition in Step 5, we note that the landing page may contain other lists of servers (mainly residing in Content Delivery Networks) where some of the static objects are downloaded and a complete HTML page is shown to the user. The mobile *apps* developers agree on integrating ads in mobile *apps* and the ads are served according to various rules set by the ad networks, such as to fill up their advertising space, and/or obtaining *profiling* information for *targeting*. Additionally, the ads refreshment intervals, mechanisms used to deliver ads (push/pull techniques), the strategy adopted after an ad is being clicked, and click-through rates, etc., are also defined by the ad networks.

Overall, the ad networks are complex systems, with multiple participants and various mechanisms to deliver ads [8, 35]. In order to understand and evaluate privacy issues, it is important to have an understanding of the ad distribution mechanisms (the selection of ads from the ad networks’ ads pool for delivery to users) and how these relate to individual user’s interest profiles and activities.

As the advertising networks are also closed systems with no external transparency, all the measurements need to be done indirectly, with complex factors including Real Time Bidding (RTB) making this non-trivial.

### Understanding ad network’s operation

The advertising networks provide an SDK for integrating ads within the mobile *apps*, while securing the low-level implementation details. The ad networks also provide the rules for embedding ads into the mobile *apps*, the ad delivery mechanism, they determine the amount of time an ad is displayed on the user’s screen and how often an ad is presented to the user. The common type of ad is the flyer, which is shown to the user either at the top or the bottom of the device’s screen (the entire screen may also be used for the duration of the ad presentation). These flyers are composed of text, images, and the JavaScript codes.

The ad presentation workflow of Google AdMob can be observed on the previously described in Fig. 1. This figure shows the flow of information for an ad request made by an *app* to AdMob, along with the action triggered after the user clicks that particular ad. The figure also shows the HTTP requests and the servers (i.e. Content Delivery Network (CDN) or ad servers) used by AdMob. Furthermore, several entities/services and a number of HTTP requests to interact with the ad servers and user agent are also shown in this figure.

### Ad traffic analysis

#### Extracting ad traffic

Recall that the mobile ad network includes interactions between different entities during the ad presentation and after an ad click (by the user) to download the actual contents of the ad, as shown in Figs. 1 and 5. Specifically, these entities are: the products, the ad agencies attempting ad campaigns for the products, ad networks delivering ads, the publishers developing and publishing mobile *apps* and the end customer to whom ads are delivered [23]. It is likely, when it comes to large publishers, that both the publishers and advertisers may have their own ad servers; in this case, some publishers may include a specific pool of ads on the advertisers’ side and, at the same time, maintain their own ad pool [24]. By having redundant ad sources, the publishers can safeguard against service disruption and ensure their revenue stream. In line with this approach, the end-user traffic may traverse several ad networks, from publishers to the advertisers, to access ads.

#### Ads traffic identification

As per Sect. 4.1, the advertising network is a closed system, therefore necessitating an indirect approach to identifying ad traffic. This can be performed by first capturing the traces from the *apps* that execute and download the ad traffic and then investigating the traffic characteristics. Characterising the ad traffic can provide information about the approaches used by multiple publishers, various mechanisms used to deliver ads by the publishers, the use of different ad servers, and the ad networks themselves [36]. Similarly, this will also assist in identifying any *analytics* traffic used by the ad networks to *target* users with relevant ads.

Analysis of the traffic traces resulted in classifying these as traffic related to (i) ad networks, (ii) the actual web traffic related to ad, (iii) traffic related to CDNs, (iv) *analytics* traffic, (v) tracking traffic, (vi) ad auctions in RTB, and (vii) statistical information about *apps* usage or developer’s statistics, and (viii) traffic exchange during and after an ad click.

#### Mobile versus in-browser ads traffic analysis

We note that there are notable differences in collecting and analysing the mobile and *in-browser* user’s ad/data traffic for the ad delivery mechanism, in order to *target* users. Analysing the mobile ad traffic requires the ability to derive a comprehensive set of rules to study the ad delivery behaviours (as various ad networks adopt their own formats for serving ads, as mentioned above), catalogue connection flows, and classify ads categorisation. Furthermore, the ad delivery mechanisms are not publicly available, hence analysing mobile targeted ads poses additional challenges due to inadequate information. The *in-browser* ad delivery mechanism can be customised^19^ to receive ads that are tailored to a specific profiling interests [37, 38].

For the *in-app* ads delivery [8, 9, 39–41], an ad network may use different information to infer users’ interests, in particular, the installed applications along with the device identifier to profile users and to personalise ads pool to be delivered. In addition, the *analytics* companies evaluate the user *profiling* [42] for *in-browser* ads via different means such as browsing history, web searches, etc., that is carried out using configured cookies and consequently *target* users with personalised ads. However, in *in-app* ad-context, this information might be missing, or altogether not permitted by the OS, as the notion of user permissions may easily prevent access to data out of the *apps* environment.

### Characterisation of *in-app* advertisements

We note that there are very few research works available on the characterisation of *in-app* (mobile) targeted ads. Prior research works have determined the large extent to which *apps* collect user’s private information [23], the potential consequences of presented ads to user’s privacy [6] and the increased utilisation of mobile device resources [24, 43]. In our previous study [27] (and in [44]), we observe that various information exchanged with the ad networks and the level of ads *targeting* are based on communicated information, similarly, we [10] investigate the installed *apps* for leaking targeted user data. To address data leakage issues, there are several works that propose the privacy-preserving [37, 38, 45] and resource-efficient mobile advertising systems [24, 43]. The primary focus of mobile ads characterisation is on measuring the efficiency of *targeted* advertising and to evaluate improved performance of *targeted* advertising for *click-through rates* [31]. However, we note that there are limited insights about evaluating the effectiveness of *targeting* mobile advertising that will ultimately determine the magnitude of various issues, e.g. operational efficiency, including the loss of privacy.

There are a number of reasons why the existing *in-browser* [6, 31, 37, 38, 46–51] ads characterisation approaches on *targeted* advertisements cannot be directly applied to the evaluation of *in-app* *targeted* ads: *First*, there may be various factors for *in-app* ads targeting that go beyond what is considered for *in-browser* ads, e.g. the context of mobile *apps* installed on the user device, their utilisation behaviour (e.g. heavy gamers may receive specific ads). *Second*, the ads classification may require unifying of the mobile market place(s) and traditional online environments, since the ads may relate to the advertisers’ businesses (i.e. the merchant websites) and to other *apps* that may be purchased (or freely available) and downloaded to mobile devices. *Third*, the methodology for collecting information about *in-app* ads is different than for the *in-browser* ads, since the ad delivery process for *in-app* ads changes with every selected ad network. *Finally*, *apps* come with pre-defined *apps* permissions to use certain resources, hence, allowing *apps* to filter part of the information to be provided to the ad network.

**Fig. 6:**
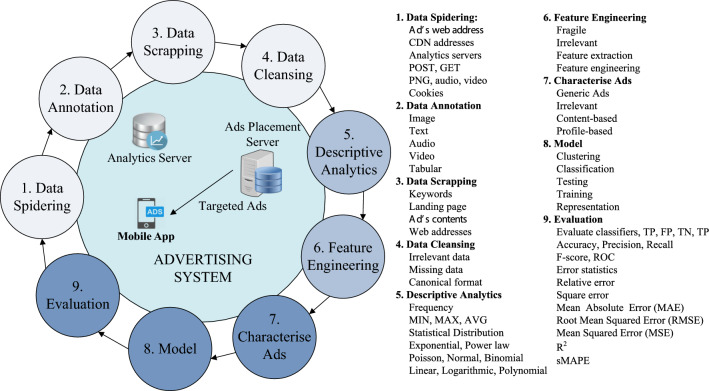
The process of ads characterisation for both *in-app* and *in-browser* targeted ads. Various steps for preparing data for ads characterisation are given from ‘1’ through ‘6’, ads characterisation is done via ‘7’, various models can be applied given in ‘8’, finally, various evaluation metrics are given in ‘9’

Figure 6 shows the lifecycle of characterising the ads traffic within the advertising system, both for *in-app* and *in-browser* *targeted* ads; various data scrapping elements and statistical measures are also shown on the right side of this figure.

In the following section, we discuss works on the characterisation of *in-app* and *in-browser* targeted ads.

#### In-app (mobile) ads

A number of studies characterise various features of *in-app* ad traffic with the focus on *targeted* advertising. The MAdScope [44] and [27] collects data from a number of *apps*, probes the ad network to characterise its *targeting* mechanism and reports the *targeted* advertising using profiles of specific interests and preferences. The authors in [43] analyse the ads harvested from 100+ nodes deployed at different geographic locations and 20 Android-based phones and calculated the feasibility of caching and pre-fetching of ads. The authors in [24] characterise the mobile ad traffic from a number of dimensions, such as the overall traffic, the traffic frequency, and the traffic implications in terms of, using well-known techniques of pre-fetching and caching, energy and network signalling overhead. This analysis is based on data collected from a major European mobile carrier with over three million subscribers. The [52] shows similar results based on the traces collected from more than 1,700 iPhone and Windows Phone users.

The authors in [53] show that *apps* from the same category share similar data patterns, such as geographic coverage, access time, set of users, etc., and follow unique temporal patterns e.g. entertainment *apps* are used more frequently during the night time. The [54] performs a comparative study of the data traffic generated by smartphones and traditional internet in a campus network. Another work [55] studies the cost overheads in terms of the traffic generated by smartphones that are classified into two types of overheads: the portion of the traffic related to the advertisements and the *analytics* traffic, i.e. traffic transmitted to the third-party servers for the purpose of collecting data that can be used to analyse users’ behaviour, etc. Several other works, [56–58], study *profiling* the energy consumed by smartphone *apps*.

#### In-browser ads

There are a number of works on characterising *in-browser* ads with the focus on issues associated with user privacy [48, 50]. In Ref. [6], the authors present a classification of different trackers such as cross-site, in-site cookie sharing, social media tracking, and show the dominance of *tracking* for leaking user’s privacy, by reverse engineering user’s profiles. They further propose a browser extension that helps to protect users’ privacy. Previous research works show how third parties effectively track consumers across multiple *apps* [59], the mobile devices responsible for leaking *Personally Identifiable Information* (*PII*) [60, 61], and gaining access to user’s private and sensitive information using well-defined APIs [62]. Another study [63] predicts various tracking information (such as viewed products, searches, or emails) in an arbitrary web account, by using differential correlation technique, to target users with different services, such as, products recommendation, targeted ads. Similarly, [64] investigates the ad fraud that generates spurious revenue affecting the ad agencies. In addition, other studies, such as [65], describe challenges in measuring online ad systems and [51] provides a basic understanding of characteristics and changing aspects of advertising and *targeting* approaches by various entities in an ad ecosystem.

## Privacy in mobile advertising: challenges

Privacy violations involve various harmful activities; following are a few privacy violations examples; a company selling the personal information of its customers despite promising not to sell, a government detecting citizen’s electricity pattern usage during the day, a grocery store scanning the list of purchased goods to find food consumption, a newspaper disclosing the name of a rape victim, etc. In brief, the discussion of privacy appeals to people’s fear and anxieties when personal information is gathered by companies [66]. In addition, the Personally Identifiable Information (PII) is the ‘information that can be used to distinguish or trace an individual’s identity^20^’, which if compromised or disclosed without authorisation, may result in harm, embarrassment, inconvenience, or unfairness to an individual. Recall that the user *profiling* and *targeted* advertising potentially expose sensitive and damaging information about consumers, also demonstrated in [67–69].

Giant tech companies, e.g. Apple, have taken enhanced consumer privacy awareness initiatives to protect user privacy. E.g. Apple’s enabling of ad blockers in iOS9^21^ is a symbolic move towards giving users greater control over the presentation of the ads, though applicable only to browser-based ads. However, we note that this would significantly affect Google’s services since Google’s services are now based on *Web & App* activity^22^.

Therefore, *targeted* advertising needs to be able to effectively serve relevant ads to appropriate users while protecting users’ privacy. In particular, it needs to enable private user *profiling* and *targeted* ads without exposing user interests to the adverting or third-party ad/tracking companies. It additionally needs to include a private *billing* process that would update the advertising network in regard to the retrieved/clicked ads in a privacy-preserving manner.

### Privacy attacks

We focus on the main types of privacy attacks that we believe are most relevant to the ad networks: unintended privacy loss, privacy leakage via cross-linking or de-anonymisation, and privacy leakage via statistical inference. We note that in all these scenarios, generally the user does not oppose the *profiling* process and is willing to receive behavioural services, e.g. *targeted* ads, on selected topics of interest, but does not wish for specific parts of their profile (*attributes*), based on the *apps* contexts (s)he considers private, to be known to the associated *analytics* companies or any third party, or to be used for personalised services.

#### Unintended privacy loss

In this case, users voluntarily provide personal information, e.g. to OSNs, or users authorise third-party services to access personal information, e.g. third-party library tracking in mobile *apps*; however, users may not be aware how the information is used and what are the potential privacy risks.

#### Privacy leakage via cross-linking or de-anonymisation

The user profile is (legitimately) determined by the *analytics* network ( e.g. [8–10]) by cross-linking private information or via de-anonymisation. In the former case, the *analytics* services aggregate user data from sources that supposedly come as a result of users (willingly) sharing their data with various entities that provide them with personalised services. In the latter case, data owners may release processed (according to a selected privacy-preserving technique) personal information, data may be purchased by advertisers or the processed (for privacy) data may be freely available on various websites^23^. The processed data is then used by attackers to disclose the identity of the data owners by cross-linking it to external data sources, i.e. using background knowledge [10].

#### Privacy leakage via statistical inference

The statistical inference, i.e. an *indirect* attack on user privacy, involves a third party profiling users based on their behaviour, to provide personalised services. E.g. the advertising systems like Google or Flurry monitor the ad traffic [10, 27] sent to mobile devices and infer the user profile based on their *targeted* ads. The profiling attributes that are sensitive to the users are considered private information, e.g. political or religious views, sexual orientation, etc.

### Ad traffic analysis for evaluating privacy leakage

Several works investigate the mobile targeted ad traffic from the point of view of privacy and security concerns. The AdRisk [3], an automated tool, analyses 100 *ad libraries* and studies the potential security and privacy leakages of those libraries. The *ad libraries* involve the resource permissions, permission probing and JavaScript linkages, and dynamic code loading. Parallel to this work, [70] examines privacy vulnerabilities in the Android-based *ad libraries*. The authors categorise the permissions acquired by the ad libraries into *optional*, *required*, or *un-acknowledged* and investigate privacy concerns such as how user’s data is communicated in the ad requests. The authors in [71] analyse the privacy policy for collecting *in-app* data and study how the integrated *analytics libraries* collect the user.

Other works [72, 73] study the risks due to the lack of separate working mechanisms among Android *apps* and ad libraries and recommend methods for splitting their functionality. The authors in [23] monitor the flow of data between the ad services and 250K Android *apps* and demonstrate that currently proposed privacy-protecting mechanisms are not effective. They propose a market-aware privacy-enabling framework to achieve the symmetry between the developer’s revenue and the user’s privacy. Another work [74] carried out a longitudinal study of the behaviour of Android *ad libraries*, in 114K free *apps*, concerning the permissions allocated to various *ad libraries* over time. The authors found that over several years, the use of most of the permissions has increased, raising privacy and security concerns.

There have been several other works that explore web advertisements in different ways, i.e. from the monetary perspective [31, 75], from the perspective of privacy of the information of users [76], from privacy information leakage, and to propose methods to protect user data [77, 78], and E-Commerce [79]. Similarly, the web ad networks have also been investigated [80] - regarding the information communicated on the network level, the network layer servers, and from the point of the content domains involved in such a system.

### Inference of private information

In recent years, several works [81–89] have shown that it is possible to infer hidden private information of subscribers of online services such as age, gender, relationship status, etc., from their generated content. The authors in [85] analysed the contents of 71K blogs at blogger.com and were able to accurately infer the gender and age of the bloggers. This was achieved by identifying certain unique features of an individual’s writing style such as parts-of-speech, function words, hyperlinks and various content, such as simple content words and the special classes of words taken from the handcrafted LIWC (Linguistic Inquiry and Word Count) [90] categories.

Another study [81] has shown that the age demographics of Facebook users can be predicted by analysing the language used in status update messages (both using *apps* and browsers). Similar inferences have been made for IMDB users based on their movie reviews [86]. Another work [88] predicts age, gender, religion, and political views of users from the queries using models trained from Facebook’s ‘Like’ feature. In [83], the authors examined the client-side browsing history of 250,000 users and inferred various private attributes including age, gender, race, education, and income. Furthermore, several studies [91–93] have demonstrated that sensitive attributes of user populations in online social networks can be inferred based on their social links, group memberships, and the privacy policy settings of their friends [94].

### User information extraction

We experimentally evaluate [10] the approaches to extracting user profiles determined by the mobile *analytics* services based on the device identifier of target users; this method was demonstrated for two major companies, i.e. Google *analytics* and Flurry, in the Android-based environment. The user profile, i.e. information collected or determined by these two *analytics* services, consists of personally identifiable information including the unique device ID, user demographics, their interests inferred from the *app* usage, etc.

A crucial technique to extract user profiles from the *analytics* services (we mainly target Google and Flurry *analytics* services) is to first impersonate the victim’s identity. Following, for ***Case 1 Google analytics***, we fetch user profiles from a spoofed device; here the private user profile is simply presented by the Google service in the form of ads preference settings. For ***Case 2 Flurry analytics***, we provide the target’s identity to a controlled *analytics* *app*, which impacts the Flurry consumer analysis report. The adversary uses this report to extract the legitimate target user profile.

In the following subsection, we first demonstrate how to obtain and spoof a device’s identity; subsequently, we present how to extract user profiles from Google and Flurry services.

#### Information extraction via user profiles from Google

Google analytic allows users to view and control their ads preferences^24^, e.g. to *update/delete* interests or to *opt-out*. The user interest profiles are associated with an advertising ID. Hence, to impersonate users’ profiles, an adversary can easily access the victim’s profile on a spoofed device.

We note that there are at least two possible ways that an adversary can capture victims’ Android IDs. First, an adversary can intercept the network communication, intercept the usage reporting messages that third-party tracking APIs communicate, extract the device identifier, and to further use it for ongoing communication with the *analytics* services. Note that it is common practice to monitor the IDs of numerous users in public hotspots, e.g. airports, hospitals, etc. Similarly, in a confined area, an adversary (e.g. a colleague or employer) targeting another individual can associate the device ID to their target (e.g. employees or another colleague). During this privacy attack, we note that Google *analytics library* hashes the Android IDs in order to prevent leakage of device identifiers; however, this practice cannot stop third-party *ad libraries* to transmit private information in plain text (note that this can be effortlessly mapped to Google’s hashed device ID).

An alternative way, although may be more challenging in practice, is to obtain the target’s device identifier from any application (controlled by the adversary) that logs and exports the device’s identity information.

#### Information extraction via user profiles from Flurry

We note that it is more challenging to extract user profiles from Flurry, as there is no option to directly view or edit user’s *Interests profiles*. Moreover, the majority of smartphone users may not be aware of Flurry’s tracking activity except for the initial consent on the access to device resources.

**Fig. 7:**
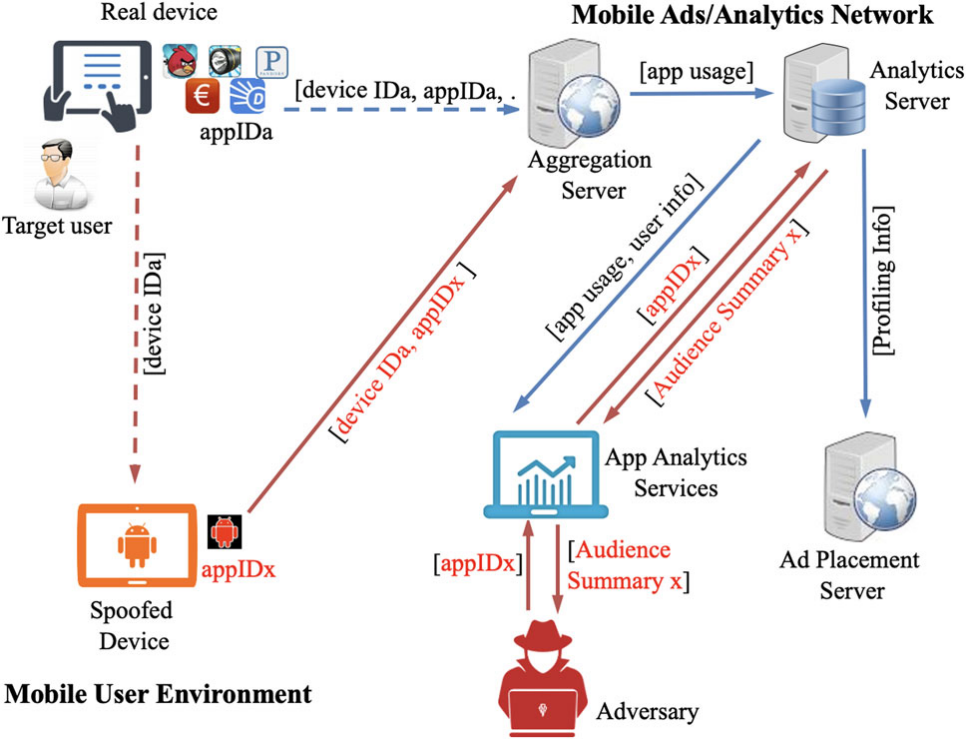
Privacy leakage attack scenario

Figure 7 shows the basic operations of our profile extraction technique within the mobile advertising ecosystem. To compromise a user’s private profile, an attacker spoofs the target device, $deviceID_a$, using another Android device or an emulator. Following, to trigger any usage reports/messages communicated to Flurry, a *bespoke* *app* with a (legitimate) $appID_x$ is used by the adversary that is installed on *spoofed* device. Henceforth, the *spoofed* device manipulates the *analytics* service into believing that $deviceID_a$ being tracked by the system. Consequently, the Flurry system grants access to all user-related private information to the adversary via the audience analysis report of legitimate $appID_x$.

Following, once the audience report from Flurry is used to target a specific user, it is easy for an adversary to extract corresponding statistics and relate them to the (legitimate) user. Furthermore, it allows the adversary to track and access all subsequent changes to the user profile. In our presented technique, since a target’s device ID is being impersonated, we can effortlessly associate a target with a ‘blank’ Flurry-monitored application.

Alternatively, via monitoring audience analysis report differences of legitimate users before and after a target ID has been spoofed, the adversary could determine an individual’s profile from an aggregated audience analysis report. Later on, the report can be added to the audience pool for personalised services. Specifically, following a series of steps; the adversary needs to take a snapshot of the audience analysis report $P_t$ at time *t*, to use the controlled Flurry-tracked application to impersonate a target’s identity, subsequently, the adversary generates another copy of the audience analysis report $P_{t+1}$ at $t+1$. Lastly, the adversary obtains the target’s profile by extracting the difference between $P_t$ and $P_{t+1}$, i.e. $\varDelta (P_t,P_{t+1})$. However, in practice, we note that the Flurry service usually updates the profiling attributes once a week, henceforth, it will take up to a week to extract a meaningful user profile.

Finally, with the *segment* feature provided by Flurry, other filters, e.g. age group, gender, and/or other developer-defined parameters can be applied to further split the *app* audience report. The adversary exploits this feature to further efficiently isolate and extract user profiles. For example, the *segment* filter ‘only show users who have Android ID value of *x*’ may be applied to generate the audience profile that contains only a particular user *x*. This particular attack is effective and is validated in the following two steps: 1. Primarily, we validate that the victim is receiving *targeted* ads in accordance with the user’s profile. We confirm this by determining that specific profiles would consistently be presented with similar ads; conversely, an update in the user profile would result in receiving unrelated ads compared to its earlier state. 2. Following, we carry out an ad influence attack over the victims’ profiles, i.e. we perturb the victims’ profiles and demonstrate that the perturbed user profiles would receive ads according to the profiling interests being modified.

### Third-party privacy threats

The third-party A &A libraries have been examined in several works, such as [3, 24, 25, 70, 95], which contribute to the understanding of mobile tracking and collecting and disseminating personal information in current mobile networks. The information stored and generated by smartphones, such as call logs, emails, contact lists, and GPS locations, is potentially highly sensitive and private to the users. In the following subsections, we discuss various means through which users’ privacy is exposed.

#### Third-party tracking

The majority of privacy concerns of smartphone users are resulting from the inadequate access control of resources within smartphones. E.g. Apple iOS and Android employ fine-grained permission mechanisms to determine the resources that could be accessed by each application. However, smartphone applications rely on users to allow access to these permissions, where users are taking risks by permitting applications with potentially malicious intent to gain access to confidential data on smartphones [96]. Similarly, the authors in [11, 97] examine privacy threats (i.e. direct and inferred information leakage) from individual’s data collected online, including the third-party ad tracking [98, 99].

Prior research works show how the third parties effectively track the consumers across multiple *apps* [59], *apps* accessing user’s private and sensitive information through well-defined APIs [62], mobile devices leaking *PII* [60, 61], inference attacks based on ads monitoring [10] and other data platform such as eXelate^25^, BlueKai^26^, and AddThis^27^ that collect, augment and resell cookies.

The authors in [100] conducted a user survey and showed that a minor proportion of users pay attention to granting access to permissions during installation and even a smaller number understand these permissions. Their results show that 42% of participants were unaware of the existing permission mechanism, only 17% of participants paid attention to permissions during *apps* installation, while only 3% of participants fully understood the meaning of permissions accessing particular resources. The authors in [3] evaluate potential privacy and security risks of information leakage in mobile ads by the embedded *libraries* in mobile applications. They studied 100,000 Android *apps* and identified 100 representative *libraries* in 52.1% of *apps*. Their results show that the existing *ad libraries* collect private information, which is mainly used for legitimate *targeting* purposes (e.g. user location), whereas the purpose for collecting reminder data is hardly justified, i.e. users’ call logs, contact details, bookmarks, the user installed *apps*. Additionally, they identify various *libraries* that use insecure public networks to directly collect user data, which is an additional serious security risk. A number of works [101–103] identify the security risks on the Android system by disassembling the applications and tracking the flow of various methods defined within various programmed classes.

Several works aim to protect privacy by assisting users to manage permissions and resource access. The authors in [104] propose to check the manifest^28^ files of installed mobile *apps* against the permission assignment policy and block those that request certain potentially unsafe permissions. In MockDroid [105], the authors propose to track the resource access and rewrite privacy-sensitive API calls, to block information communicated outside the mobile phones. Similarly, the AppFence [106] adds taint-tracking to further improve this approach, thereby allowing more refined permission policies.

#### Re-identification of sensitive information

Re-identification involves service personalisation based on pervasive spatial and temporal user information that has already been collected, e.g. previously visited locations. The users are profiled and later on provided with additional offers based on their interests, such as recommending places to visit, or people to connect to. There have been a number of research works demonstrating how users may be identified based on the re-identification technique. For instance, the authors in [107] analyse U.S. Census data and show that every 20 individuals, on average, share the same work or home locations, while they were able to uniquely identify 5% of the people using the home-work location pairs. Another related work [108] uniquely identifies US mobile phone users, by generalising the top *N* home-work location pairs. The authors use location information to derive quasi-identifiers for the re-identification of users. Similarly, a number of research works e.g. [109–111], raise privacy issues in publishing sensitive information and focus on theoretical analysis of *obfuscation* algorithms to protect user privacy.

### Quantifying privacy algorithms

Quantifying privacy is an important and challenging task as it is important to evaluate the level of privacy protection achieved. It is difficult to formulate a generic metric for quantifying privacy that applies to different contexts due to several types of privacy threats. It is also the different solutions, i.e. specific techniques (not necessarily threats) that contain their unique privacy metrics, which are not cross-comparable.

For instance, the proposal for fulfilling the privacy requirements using *k*-anonymity, first proposed in [112], requires that each equivalence class i.e. set of records that are indistinguishable from each other concerning certain identifying attributes must have a minimum of *k* records [113]. Another study [114] reveals that satisfying the privacy requirements for *k*-anonymity cannot always prevent attribute disclosures mainly for two reasons. First, an adversary can easily discover private sensitive attributes when these exhibit a low level of diversity. Second, *k*-anonymity is not resistant to privacy attacks against attackers that use background knowledge. [114] proposes an *l*-diversity privacy protection mechanism against such attacks and evaluates its practicality both formally and using experiment evaluations. Another work [115] evaluates the limitation of *l*-diversity and proposes *t*-closeness, suggesting the distribution of sensitive attributes in an equivalence class must be close to the distribution of attributes in the entire set of data, i.e. the distance between two distributions should not be more than the *t* threshold.

Additional techniques based on crypto mechanisms, such as PIR, provide privacy protection, for the database present on *single-server*, against the computational complexity [116, 117], *multiple-servers* for protecting privacy against colluding adversaries [118–122], or protection mechanisms [123] against combined privacy attacks that are either computationally bounded evaluations or against colluding adversaries. These techniques are discussed in detail in Appendix A.

## Privacy in mobile ads: solutions

The *direct* and *indirect* (i.e. inferred) leakages of consumers’ information have raised serious privacy concerns. A number of research works propose private *profiling* (and advertising) systems [38, 45, 124–127]. These systems do not reveal either the users’ activities or the user interest profiles to the ad network. Various mechanisms are used to accomplish these goals: Adnostic [38], Privad [125] and Re-priv [124] focus on ads *targeting* based on users’ browsing activities, and are implemented as browser extensions running the *profiling* algorithms locally in the browser. The MobiAd [45] proposes a distributed approach, specifically aimed at mobile networks. The use of *differential privacy* is advocated in *Practical Distributed Differential Privacy* (PDDP) [126] and SplitX [127], where differentially private queries are conducted over distributed user data. Altogether these works partly require the re-design or suggest entirely replacing the existing advertising systems to protect user privacy, though some solutions e.g. Adnostic, can co-exist with the current systems. Furthermore, other works based on obfuscation techniques, e.g. textitdifferential privacy, obscure user preferences; however, it may result in lower accuracy of *targeted* ads and hence result in lower revenues.

**Fig. 8:**
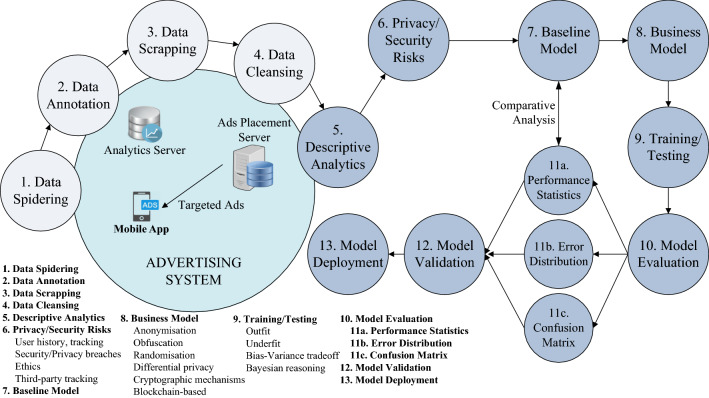
Lifecycle of proposal for privacy-preserving advertising systems for both *in-app* and *in-browser* targeted ads

Figure 8 shows the lifecycle of the proposal for privacy-preserving mobile/web advertising systems; specifically starting from data collection for evaluating privacy/security risks, baseline model, and proposed business model for preserving user’s privacy, finally model evaluation and its comparison with the baseline model. Various data scrapping elements, statistical measures, and privacy-preserving techniques are also shown in this figure.

An important thing in the development of a private advertising system is that the consumers’ trust in the privacy of mobile ads is positively related to their willingness to accept mobile advertising [128, 129]. The AdChoices^29^ program (a self-regulation program implemented by the American ad industry), states that, to control ads from third-party networks, consumer could *opt-out* of *targeted* ads through online choices. However, another study [130] examines that *opt-out* causes presenting less relevant ads, lowers the click-through rates, and generates less revenue (up to 52% lower) than allowing the *targeted* advertising while disabling the *opt-out* option. Furthermore, the authors determined that only 0.23% of American consumers requested the ad impressions.

### Private ad ecosystems

Several generic privacy-preserving solutions address the negative impact of *targeted* advertising. Solutions for web browsing based on *anonymity* include the use of Tor [131], or disabling cookies [132]. These accomplish the goal of preventing user tracking; however, they also prevent any user (profile-based) service personalisation, which may be a desirable feature for many users despite their privacy concerns.

Research proposals to enable privacy-preserving advertising have been more focused on web browsing, as the dominant advertising media, e.g. [38, 39, 125, 127, 133], propose to use locally derived user profiles. In particular, Privad [125] and Adnostic [38] download a wide range of ads from the associated ad network and locally (browsers or on mobile devices) select and present ads that match the user’s profile. On the other hand, there are a smaller number of works that address privacy for mobile advertising, with representative works, e.g. [8, 9, 36, 40, 45, 134, 135], suggest the *app*-based user *profiling*, locally store on a mobile device. The proposal presented in [8] is based on various mechanisms of PIR, and it complements the existing advertising system. It is conceptually closest to [134], which uses Oblivious RAM (ORAM) to perform Private Information Retrieval (PIR) on secure co-processor hardware. However, compared to [8], it relies on specific (secure) hardware to enable PIR, which may limit its applicability in a general setting.

### Data masking, generalisation, obfuscation and randomisation

In this subsection, we present solutions that are based on privacy techniques of data masking and generalisation, data randomisation, and obfuscation.

#### Data masking and generalisation

The simplest and most straightforward way to endeavour to *anonymise* data includes masking or removing data fields (attributes) that comprise *PII*. These include direct identifiers like names and addresses, and quasi-identifiers (QIDs) such as gender and zip code, or an IP address; the latter can be used to uniquely identify individuals. It is assumed that the remainder of the information is not identifying and therefore not a threat to privacy (although it contains information about individuals, e.g. their interests, shopping patterns, etc.). A second approach is to generalise QIDs, e.g. by grouping them into a higher hierarchical category (e.g. locations into postcodes); this can also be accomplished according to specified *generalisation* rules. Various mechanisms that deal with selected QIDs according to pre-determined privacy rules include *k*-anonymity [136] and its variants like *l*-diversity [114] and *t*-closeness [115]. The basic technique, *k*-anonymity (description of *k*-anonymity is presented in 1), modifies (*generalise*) individual user records so that they can be grouped into identical (and therefore indistinguishable) groups of *k*, or additionally apply more complex rules (*l*-diversity and *t*-closeness).

We note that according to the privacy terminology defined in [137], the term pseudonymity should be used in place of anonymity for processed data that may be used to re-identify users. We also note that both terms are used in various privacy documents, e.g. Google privacy terms^30^, IBM privacy^31^.

Several proposals promote locally (browser-based or mobile devices) derived user profiling, where the user’s interests are *generalised* and/or partially removed based on user’s privacy preferences; following, these preferences are forwarded to the ad server that selects appropriate ads for the clients. Furthermore, the removal of direct identifiers includes user IDs (replacing with temporary IDs) or hiding the network address (e.g. using Tor [131]). However, the ad networks ecosystem would be effectively disabled if only the most obvious privacy technique is applied without introducing additional *profiling* and *targeting* oriented features. Therefore, we only mention representative solutions from this category and concentrate on the privacy-preserving mechanisms that enable *targeted* ads.

Several other works [43, 45, 125], in addition to privacy, use cache mechanism for achieving network bandwidth efficiency for ad delivery. Furthermore, such techniques have been demonstrated to be vulnerable to composition attacks [138], and can be reversed (with individual users identified) when auxiliary information is available (e.g. from online social networks or other publicly available sources) [139, 140].

In Adnostic [38], each time a webpage (containing ads) is visited by the user; the client software receives a set of generic ads, randomly chosen by the broker. The most appropriate ads are then selected locally, by the client, for presentation to the user; this is based on the locally stored user profile. We have categorised this work as a *generalisation* mechanism as the served ads are generic (non-personalised), although it could arguably be considered under the *randomisation* techniques. We note that in [38] the user’s privacy (visited pages or ad clicks) is not protected by the broker.

In Privad [37, 125], a local, user profile is generated by the Privad client and then *generalised* before sending to the ads broker in the process of requesting (broadly) relevant ads. All communication with the broker is done through the dealer, which effectively performs the functions of an *anonymising* proxy; the additional protection is delivered by encrypting all traffic, thus protecting the user’s privacy from the dealer. The proposed system also includes monitoring of the client software to detect whether any information is sent to the broker using, e.g. a covert channel. Similarly, in MobiAd [45], the authors propose a combination of peer-to-peer mechanisms that aggregates information from users and only presents the aggregate (*generalised* activity) to the ad provider, for both ad impressions and clicks. Caching is utilised to improve efficiency and Delay tolerant networking for forwarding the information to the ad network. Similarly, another work [141] proposes combining users’ interests via an ad hoc network, before sending them to the ad server.

Additionally, some system proposals [142] advocate the use of *anonymisation* techniques (*l*-diversity) in the *targeting* stage, where the ads are distributed to users, while utilising alternative mechanisms for *profiling*, learning and statistics gathering.

#### Obfuscation

*Obfuscation* is the process of obscuring the intended meaning of the data or communication by making the message difficult to understand.

In the scenario of an advertising system, recall that the user privacy is mainly breached for their *context*, i.e. specific use of mobile *apps* from an *app* category, and their profiling *interests* along with the ads targeting based on these interests. Hence, an important focus in implementing such mechanisms is to *obfuscate* specific profiling attributes that are selected as private (i.e. the attributes that the analytics companies may use for interest-based advertisements) and the categories of installed *apps*. For example, the user may not wish the categories of gaming or porn to be included in their profile, as these would reflect heavy use of corresponding (gaming and porn) *apps*. The *obfuscation* scenarios can be based on similar (obfuscating) *apps* or similar profiling attributes or interests customised to user’s profile [9] or randomly chosen *apps/interests* from non-private categories. An important factor is to take into consideration the extra (communication, battery, processing, usage airtime) overhead while implementing *obfuscation* mechanisms; following, it needs to present a jointly optimised framework that is cost-effective and preserves user privacy for profiling, temporal *apps* usage behavioural patterns and interest-based ads targeting.

A recent work [143] carries out comprehensive investigation over the use of *obfuscation* analysing 1.7 million free Android *apps* from Google Play Store to uncover various *obfuscation* techniques, finding that only 24.92% of *apps* are obfuscated by the developer. There are several *obfuscation* mechanisms for protecting private information, such as the *obfuscation* method presented in [144] that evaluates different classifiers and *obfuscation* methods including greedy, sampled and random choices of obfuscating items. They evaluate the impact of *obfuscation*, assuming a prior knowledge of the classifiers used for the inference attacks, on the utility of recommendations in a movie recommender system. A practical approach to achieving privacy [145], which is based on the theoretical framework presented in [146], is to distort the view of the data before making it publicly available while guaranteeing the utility of the data. Similarly, [147] proposes an algorithm for publishing partial data that is safe against malicious attacks where an adversary can do the inference attacks using the association rule in publicly published data.

Another work, ‘ProfileGuard’ [40], and its extension [9] propose an *app*-based profile *obfuscation* mechanism to eliminate prevailing dominant private interest categories. The authors investigate insights to Google AdMob *profiling* rules, e.g. deterministically showing individual *apps* map to user’s interests within their profile and that a *stable* user profile requires a certain level of activity. To prove this, they investigate Android *apps* and carry out a wide range of experimental evaluations for several months. The authors suggest various *obfuscation* mechanisms, e.g. *similarity* with the installed *apps*, *bespoke* (customised to user profile) and *bespoke++* (i.e. *resource-aware*) strategies. Furthermore, to demonstrate its feasibility, the authors implement a an automated POC ‘ProfileGuard’ *app* *obfuscation* mechanism.

Following, we provide an overview of prior work in both *randomisation* (generic noisy techniques) and *differentially private* mechanisms.

#### Randomisation

In the *randomisation* methods, noise is added to distort the user’s data. Noise can either be added to data values (e.g. movie ratings or location GPS coordinates), or, more applicable to *profiling* and user *targeting*, noise is in the form of new data (e.g. additional websites that the user would not have visited normally are generated by a browser extension [148]), added in order to mask the true values of the records (browsing history). We note that [148] protects the privacy of user’s browsing interests but does not allow (privacy-preserving) *profiling* or selection of appropriate *targeted* ads.

The idea behind noise addition is that specific information about a user’s activities can no longer be recovered, while the aggregate data still contains sufficient statistical accuracy so that it can be useful for analysis (e.g. of trends). Several research works focus on generic noisy techniques, e.g. [149] proposed creating a randomly independent set of data and adding it to existing data using a uniform distribution. Other works, e.g. [150], improve this technique, whereas [151] identified the shortcomings of their approach where the additional noisy data may be removed using data analysis and recover the original data sets.

Another work [152] proposed a novel noisy technique for web searches that provide privacy-preserving personalisation. They use the ‘Bloom’ cookie to replace the locally derived profile with its noisy version, generated via Bloom filters [153], which is an efficient data structure. The authors also evaluate the trade-off of privacy against personalisation.

### Differential privacy

The *differential privacy*^32^ was introduced in [154], to mathematically determine the privacy loss associated with any released data or *transcript* drawn from a database. Two datasets $D_1$ and $D_2$ differ in at most one element given that one dataset is the subset of the other with a larger database containing only one additional row e.g. $D_2$ can be obtained from $D_1$ by adding or removing a single user. Hence, a *randomised* function *K* gives *differential privacy* for the two data sets $D_1$ and $D_2$ as: ${{\mathop {\textrm{P}}\nolimits } _r}\left[ {K\left( {{D_1}} \right) \in S} \right] \le \exp \left( \varepsilon \right) \times {{\mathop {\textrm{P}}\nolimits } _r}\left[ {K\left( {{D_2}} \right) \in S} \right]$. We refer readers to [155] for deeper understanding of *differential privacy* and its algorithms.

*Differential privacy* is vastly used in the literature for *anonymisation* e.g. a recent initiative to address the privacy concerns by recommending usage of *differential privacy* [156] to illustrate some of the shortcomings of direct contact-tracing systems. Google has recently published a *Google COVID-19 Community Mobility Reports*^33^ to help public health authorities understand the mobility trends over time across different categories of places, such as retail, recreation, groceries, etc., in response to imposed policies aimed at combating COVID-19 pandemic. The authors in [157] use *differential privacy* to publish statistical information of two-dimensional location data to ensure location privacy. Other works, such as [158, 159], partition data dimensions to minimise the amount of noise, and in order to achieve higher privacy accuracy, by using *differential privacy* in response to the given set of queries.

*Differential privacy* [160] has, in recent years, resulted in several systems works that advocate the practicality of this, previously predominantly theoretical research field. The authors in [126] propose to use data aggregation techniques over distributed user data for *differentially private* statistical queries. They assume *honest-but-curious* proxy between the data aggregator component and clients for secure communication, in addition to, authentication, and confidential traffic using TLS [161]. Furthermore, they use cryptography to provide user privacy. Similarly, the SplitX [127] relies on intermediate nodes to provide *differential privacy* guarantees of user data. The intermediate nodes process the user data and forward it to the data aggregator and client, which locally stores their data. Other works propose distributed *differential privacy* [162] and [163].

### Cryptographic mechanisms

A number of different cryptographic mechanisms have been proposed in the context of *profiling* and *targeted* advertising or, more broadly, search engines and recommender systems. These include: Private Information Retrieval (PIR), Homomorphic encryption, Multi-party Computing (MPC), Blockchain-based solutions.

#### Private Information Retrieval (PIR)

Private Information retrieval (PIR) [117, 118, 123, 164–166], is the ability to query a database successfully without the database server discovering which record(s) of the database was retrieved or the user was interested in. Discussion of various PIR mechanisms along with their comparison is provided in Appendix A.

The ObliviAd proposal [134] uses a PIR solution based on bespoke hardware (secure coprocessor), which enables on-the-fly retrieval of ads. The authors propose the use of the Oblivious RAM (ORAM) model, where the processor is a ‘black box’, with all internal operations, storage, and processor state being unobservable externally. ORAM storage data structure comprises entries that include a combination of keywords and a corresponding ad (multiple ads result in multiple entries). The accounting and *billing* are secured via the use of using electronic tokens (and mixing [167, 168]). More generally, a system that enables private e-commerce using PIR was investigated in [119], with tiered pricing with record level granularity supported via the use of the proposed Priced Symmetric PIR (PS-PIR) scheme. Multiple sellers and distributed accounting and *billing* are also supported by the system.

In one of our previous works [8], we propose a PIR-based mobile private advertising system. Our main motivation for using *information-theoretic* (threshold) PIR, as opposed to other solutions, e.g. Oblivious Transfer [169, 170], was the lower communication and computation overhead of these schemes, highly relevant in a mobile environment.

We note that, in conjunction with *obfuscation*, e.g. in [162, 163], or *generalisation* [38] techniques, the cryptographic solutions can also be used to partly provide the system functionality.

#### Zero knowledge proof (ZKP) and mixing

Zero-knowledge proofs [171–174] and *mixing* [175] are commonly used as components of the privacy solutions. ZKP is a cryptographic commitment scheme by which one party (the *prover*) can prove to another party (the *verifier*) that they know a value *x*, without conveying any information apart from the fact that they know the value *x*. An example of *Mixing*, called *mixnet* [167], based on cryptography and permutation, was introduced to achieve anonymity in network communication. It creates a hard-to-trace communication by using a chain of proxy servers, called *mixes*, which takes messages from multiple senders, shuffles, and sends them back in random order to the destination, hence breaking the link between source and destination and making it harder for eavesdroppers to trace end-to-end communications. A number of robust, threshold mix networks have appeared in the literature [168, 176–181]. The popular Tor browser [131] can also be considered as a type of mixed network.

Another work [126] uses the probabilistic Goldwasser-Micali cryptosystem [182] cryptographic mechanism to combine client’s data that is already modified using *differential privacy*. This work is further extended [127] where the authors use an XOR-based cryptographic mechanism to provide anonymity and unlinkability to analysis clients’ *differentially private* data. Another cryptography technique, i.e. *mixing* [167, 168] is commonly used to anonymise data [134, 183] where *mix* intermediary servers are used to encrypt the user data.

Our proposal for private *billing* for ads in [8] uses a combination of *ZKP* and *Polynomial commitment* (see discussion of these techniques in Appendix B).

**Fig. 9:**
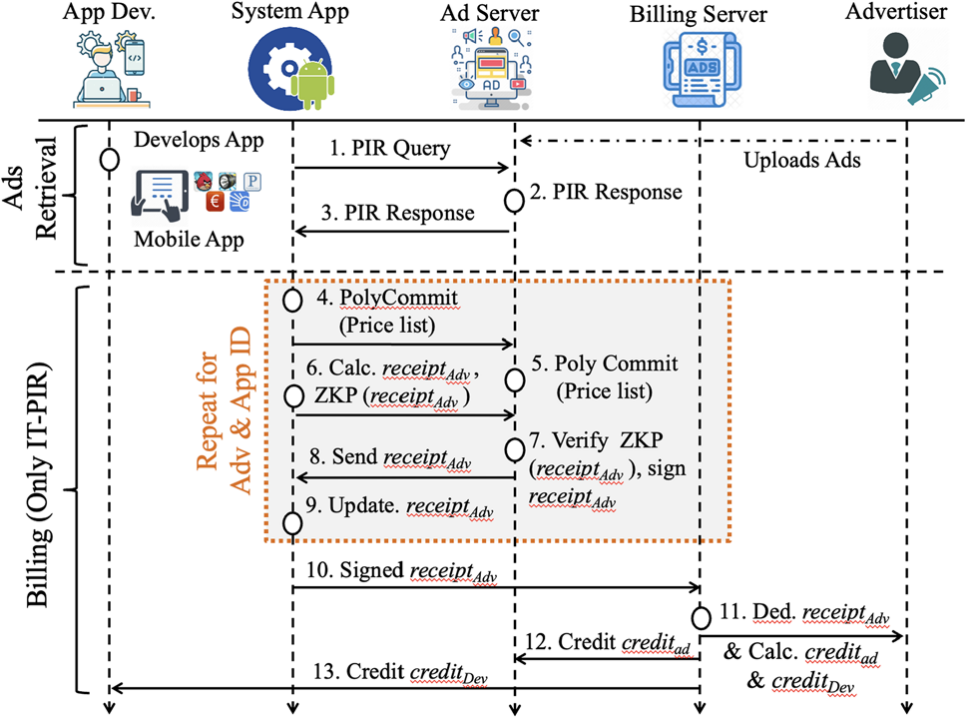
The work flow for Ads retrieval and billing for *ad presentations* and *ad clicks*

An overview of the proposal is presented in Fig. 9.The following information is assumed to be available to the *client* (software e.g. the AdMob SDK that is integrated in mobile *apps* for requesting ads and tracking user’s activity) for the entire set of ads available in the database: the *Ad* index *m*, *Ad* category ${\varPhi _i}$, *price tags* $C_T^{prs}$ and $C_T^{clk}$, respectively, for *ad presentations* and *ad clicks*, and and the *Advertiser ID* $I{D_{Adv}}$.

This private *billing* mechanism consists of two parts: the workflow for retrieving ads (Step 1–3) and private *billing* (Step 4–13). In Step 2, the Ad server evaluates the PIR response and sends it to the *client*; following, the *client* decodes the PIR response (as shown in Step 3) and subsequently forwards the retrieved ads to mobile *apps*.

The *billing* process follows the completion of the *ads presentation* (or *ad click*) processes. The *client* calculates the *receipt* locally, consisting of various components to verify the following: (a) price tier for presented ad or ad clicks; (b) the $I{D_{Adv}}$ (used for price deduction from advertiser, as shown in Step 11 of Fig. 9); and (c) the application ID (helpful for price credit to *App Developer*, i.e. Step 13). This *billing* mechanism is based on PS-PIR [119], proposed for *e-commerce*. In addition, we note that this *billing* mechanism is applicable only to single ad requests with no impact on privacy.

#### Homomorphic encryption

Homomorphic encryption [184] is a form of encryption that allows specific types of computations to be carried out on ciphertext, without decrypting it first, and generates an encrypted result that, when decrypted, matches the result of operations performed on the plaintext.

Adnostic [38] uses a combination of homomorphic encryption and zero-knowledge proof to enable accounting and private *billing* in an advertising system. The user is effectively protected where the publisher (e.g. a website that posts ads) and the advertisers (ads owner) gain no knowledge about the ads being viewed by the user. The authors in [162] also combine *differential privacy* with a homomorphic cryptosystem, to achieve privacy in a more generic setting of private data aggregation of distributed data. Similarly, Shi et al. [163] also use a version of homomorphic techniques to enable private computing of sums based on distributed time-series data by a non-trusted aggregator.

The authors in [185] present privacy-preserving recommendations using Partially Homomorphic Encryption (PHE) along with secure multi-party computation protocols. Specifically, the user’s private data is encrypted via PHE, this way the recommender cannot use their original data while still being able to generate a private recommendation, which is uploaded to the recommender system; following, the recommender runs a cryptographic protocol offline with a third party to generate personalised recommendations. This proposal also achieves good performance by lowering the processing and communication overheads by borrowing high cryptographic computations from third-party systems. Similarly, [186] proposes a recommendation system based on the ElGamal cryptosystem (i.e. a kind of PHE), where all users actively collaborate with the recommender server privately generate recommendations for a target user. Another work [187] relies on Boneh-Goh-Nissim (BGN) homomorphic cryptosystem that adopts an additional isolated recommender server that assists users in decrypting ciphertexts whenever necessary, hence actively interacting with both recommendation and additional servers.

#### Multi-party computing (MPC)

MPC [188] is a set of cryptographic methods that allow private computing (of selected mathematical functions) on data from multiple, distributed, parties, without exposing any of the input data. The formal guarantees provided by MPC relate to both data confidentiality and the correctness of the computed result.

The authors in [183] propose the first web-based advertising system based on multi-party *information-theoretic* PIR in an *honest-but-curious multi-server* architecture. Central to their system is the choice of a negotiant function, that is used by the advertiser to select ads, starting from a user’s profile—the authors describe both a semi-private and a fully private *information-theoretic* PIR in an *honest-but-curious multi-server* architecture. They evaluate the benefits of both alternatives in regard to security, computational cost, and communication overheads.

Other cryptographic techniques include functional encryption, used in data aggregation in example real-world scenarios [189, 190].

**Fig. 10:**
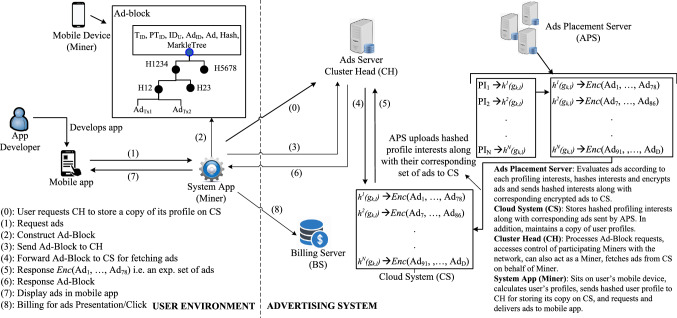
A framework for secure user *profiling* and Blockchain-based *targeted* advertising system for *in-app* mobile ads [36]. Description of various operation redirections (left side) and advertising entities (right side) is also given in this figure

### Blockchain-based advertising systems

Blockchain is a fault-tolerant distributed system that uses the distributed ledger of transactions that are shared among the participating entities and provides auditable transactions [191] being verified by participating entities in the Blockchain network. A blockchain is unalterable, i.e. once recorded, the data in any block cannot be changed without altering all the subsequent blocks; hence, it may be considered secure by design with high Byzantine fault tolerance, e.g. one-quarter of the participating nodes can be faulty but the overall system continues to operate normally.

Among the participating entities in a blockchain-based network; the *Miner* is a special node responsible for generating transactions, adding them to the pool of pending transactions, in addition, organising them into a *block* once the size of transactions reaches a specific *block size*. Subsequently, the *Miner* adds the newly processed block to the Blockchain; this process is referred to as *mining*, which follows a particular *consensus* algorithm, such as Proof of Stake (POS) [192] and Proof of Work (POW) [193]. These *consensus* algorithm guarantees the security of Blockchain against adversaries (e.g. malicious *Miner*). Furthermore, to achieve anonymity, the participating entities within the Blockchain network use the *Public-Private Key* pair [194]. There are various salient features that Blockchain offers, i.e. irreversible, auditable, updated near real-time, chronological, and timestamp, which, in addition, disregards the need for a central controlling authority. Hence, making it a candidate for securing individual’s privacy of an advertising network since it would restrict the communication between the mobile *apps* (e.g. mobile device acting as a *Miner*) and the analytics/ad companies.

Blockchain [195] has numerous applications, e.g. IoT [196], Healthcare [197], Banking and finance [198], Bid Data [199] etc. To the best of our knowledge, there are very few works available for Blockchain-based mobile *targeted* ads in the literature. E.g. [41] presents a decentralised *targeted* mobile coupon delivery scheme based on Blockchain. The authors in this work match the behavioural profiles that satisfy the criteria for *targeting* profile, defined by the vendor, with relevant advertisements. However, we note that this framework lacks many components of an advertising system, e.g. user profile construction, comprehensive formation of different types of Blockchain-based transactions, or other entities such as *Miner* and the *billing* process. Our recent work, *AdBlock* [36], presents a framework (in addition to Android-based POC implementation, i.e. a *Bespoke Miner*) for private user *profiling*, privately requesting ads, the *billing* mechanisms for ads presentation and clicks, a mechanism for uploading ads to the cloud, various types of advertising-specific transactions in Blockchain-based network, and methods for *access policy* for accessing various resources, e.g. accessing ads, storing mobile user profiles, etc. This framework is parented in Fig. 10. We further implement various critical components of the advertising system and experimentally evaluate its applicability; these components include constructing user profiles, encryption and decryption of profiling interests, and *access policies*. We observe that the processing delays with various operations evaluate to an acceptable processing time as that of the real-time advertising systems, also verified in Ref. [8].

In Ref. [36], we provide an alternative proposal for *ad presentations* and *clicks* with the use of mining Cryptocurrency (e.g. Bitcoin). Our main aim, other than preserving user privacy, was to include secure payment and ensure compatibility with the underlying *AdBlock* proposal [36] for a mobile advertising system utilising Blockchain.

The following notations are used in this proposal: price tags $C_{prs}^{A{d_{ID}}}$ and $C_{clk}^{A{d_{ID}}}$ for ad *presentation* and *click*; different types of *wallets*, i.e. *App Developer*’s $walle{t_{I{D_{APP}}}}$, *Advertiser*’s $walle{t_{A{D_{ID}}}}$, Billing server’s $walle{t_{BS}}$; *public-private key* ($PK+/-$) and (Bitcoin) addresses, i.e. $Ad{d_{I{D_{APP}}}},Ad{d_{A{D_{ID}}}},Ad{d_{BS}}$. It works as follows: The advertiser buys advertising *airtime*, it signs the message with the amount of Cryptocurrency with her *private key* ($PK-$), adds *Billing* server’s address, requests a transaction. Following, this request is bound to the other pending transactions and is broadcasted over the network for *mining*. Once the transaction completes, the Billing server receives its amount of Cryptocurrency in $walle{t_{BS}}$. In addition, the Miner initiates the *billing transaction* for ads *presentations* or *clicks*, respectively, by encoding the $C_{prs}^{A{d_{ID}}}$ and $C_{clk}^{A{d_{ID}}}$ price tags; this amount is then shared with $walle{t_{I{D_{APP}}}}$ and $walle{t_{A{D_{ID}}}}$ *wallets*.

**Table 1:** Summary of the *in-browser* and *in-app* advertising systems

Reference	Architecture	Mechanism	Deployment	Domain
Privad [125]	3rd-party anonymising proxy	Crypto	Browser add-on	Web
Adnostic [38]	Complements existing sys	Crypto billing	Firefox extension	
PASTE [162]	Untrusted third party	Fourier Perturbation Algo	Browser add-on	
[200]	Cookie management	User preference	Standalone	
[201]	Anonymising proxy	Differential privacy		
DNT [202]	Delay Tolerant Network	HTTP header	Browser side	
MobiAd [45]		Encryption	Mobile phone	Mobile
ObliviAd [134]	Complements existing sys	Crypto-based	Client/Server sides	
[135]		Differential privacy		
SplitX [127]		XOR-based encryption		
CAMEO [43]		Context prediction		
ProfileGuard [9, 40]		Profile Obfuscation		
[41]		Blockchain		
AdBlock [36]				
[8]	Autonomous system	Crypto-based	Standalone	

A summary of various privacy-preserving approaches, in terms of *architecture*, *mechanism*, *deployment* and *app domain*, for both *in-browser* and mobile advertising systems is provided in Table 1.

**Table 2:** Comparison of various privacy protection mechanisms for selected relevant parameters applicable to an advertising system

Parameters	Differential	Obfuscation		Cryptographic	Randomisation	Blockchain	Anonymisation
	privacy	App-based	Profile-based	mechanisms		solutions	
Apps usage behavioural privacy	No guarantee	Guaranteed	No guarantee	No guarantee	No guarantee	No guarantee	No guarantee
Profiling privacy	Yes	Yes	Yes	Yes	Yes	Yes	Yes
	Low	(Low to high)	(Low to high)		(Low to high)		
Indirect privacy exposure from targeted ads	Yes	Yes	Yes	No	Yes	No	Yes
Cost of achieving user privacy	Low	High	Low	High	Low	High	Low
Targeted ads	Yes (Lower)	Lower to not relevant ads (adjustable)	Lower to not relevant ads (adjustable)	Yes	Lower to not relevant ads (adjustable)	Yes	Yes
Trade-off b/w privacy and targeted ads	No	Yes	Yes	No	Yes	No	No
Impact on billing for targeted ads	Yes	Yes	Yes	No	Yes	No	No

### Comparison of various privacy protection mechanisms proposed of advertising systems

Table 2 presents a hypothetical comparison of privacy protection mechanisms for various parameters applicable to an advertising system, e.g. *Apps* or *Interest* profiling privacy, cost of achieving user privacy, etc. We plan to carry out a comprehensive study of these parameters (presented in Table 2) for privacy protection mechanisms in future work, to validate our hypotheses.

It can be observed that the *Obfuscation*-based mechanisms can guarantee user’s ‘apps usage behaviour privacy’ (as evident in [9, 40]) at the expense of installing and running a number of mobile *apps*. Similarly, the ‘cost’ of achieving user privacy with *Blockchain*-based solution is quite high due to its operational complexity [36, 41]. An important parameter is ‘impact on *targeted* ads’ as a results of achieving user privacy with various techniques e.g. *Crypto-based* techniques (such as PIR), *Blockchain* and *Data masking and generalisation* techniques will have no impact on *targeted* ads, alternatively, the *Differential privacy*, *Obfuscation* and *Randomisation* will have an impact on *targeted* ads and can be adjusted to achieve various trade-offs, i.e. ‘low-relevant vs. high-relevant interest-based ads’, as is also evident in [9, 10]. We note that these latter set of techniques will also have an impact on *billing* since the advertisers’ ads are shown to ‘irrelevant’ users, hence, they (advertisers) pay for airtime that is used by non-targeted audiences. Similarly, an important parameter is the ‘trade-off between *privacy* and *targeted* ads’ that can be achieved only with *Obfuscation* and the *Randomisation* techniques. In addition, the ‘indirect privacy attack to expose user privacy’ reflects the user privacy protection in regards to delivered ads; this attack will not work when *Crypto-based* techniques are used as the delivered ads are also protected, as shown in Ref. [8].

### The economic aspects of privacy

Research works also investigate the notion of compensating users for their privacy loss, rather than imposing limits on the collection and use of personal information.

Ghosh and Roth [203] studied a market for private data, using *differential privacy* as a measure of the privacy loss. The authors in [204] introduce transactional privacy, which enables the users to sell (or lease) selected personal information via an auction system. On a related topic of content personalisation and *in-browser* privacy, in RePriv [124] the authors propose a system that fits into the concept of a marketplace for private information. Their system enables controlling the level of shared (local) user profile information with the advertising networks, or, more broadly, with any online entity that aims to personalise content.

## Open research issues

In this section, we present future research directions that would complement the existing solutions to provide a fully functional privacy-preserving ad system.

### Diffusion of user tracking data

A recent shift in online advertising has enabled the advertising ecosystem to move from ad networks towards ad exchanges, where the advertisers bid on impressions being sold in RTB auctions. As a result, the A &A companies closely collaborate for exchanging user data and facilitate bidding on ad impressions and clicks [205, 206]. In addition, the RTB necessitates that A &A companies also work with publishers to help manage their relationship for ad exchange (in addition to user’s data tracking) and to optimise the ad placement (i.e. *targeted* ads) and bidding on advertiser’s behalf. This has made the online advertising operations and the advertising ecosystems themselves extremely complex.

Hence, it is important to model (in order to accurately capture the relationship between publisher and A &A companies) and evaluate the impact of RTB on the diffusion of user tracking (sensitive) data. This further requires assessing the advertising impact on the user’s contexts and *profiling* interests, which is extremely important for its applicability and scalability in the advertising scenarios. This will also help the A &A companies and publisher to effectively predict the tracker domain and to estimate their advertising revenue. Furthermore, it is necessary to ensure the privacy of user data that is collected and disseminated in a distributed fashion, i.e. users may be affiliated with different *analytics* and advertising platforms and their data may be shared across diverse publishers. Consequently, a distributed platform is required for the efficient management and sharing of distributed data among various A &A platforms and publishers. This is specifically driven by the RTB requirement to develop efficient methods for distributed and private data management.

### Complex operations of the advertising system

The complexity of online advertising poses various challenges to user privacy, processing-intensive activities, interactions with various entities (such as CDN, *analytics* servers, etc.), and their tracking capabilities. In order to reduce the complexity of the advertising systems, we envision several additional areas of research: devising processing-sensitive frameworks, limiting the direction-redirection of requests among A &A entities, opening up the user data exchange processes within the ad platform, identifying new privacy threats, and devising new protection mechanisms. Revealing the user data exchange will expose the extent to which the intermediate entities are prone to adversarial attacks. What is also required is improved knowledge of the adversary, which will contribute to the development of protection mechanisms for various kinds of privacy threats, e.g. interest-based attacks or direct privacy attacks. Note that this will further require a comparative analysis of basic and new proposals for the trade-off achieved between privacy and computing overheads of processing users’ ad retrieval requests/responses, communication bandwidth consumption, and battery consumption.

### Private user-driven mobile advertising systems

It is necessary to develop a novel user-driven private advertising platform, that can accommodate varying user interests (vis-à-vis their privacy) and the advertising system’s business interests. In addition, the assessment of user information as an inherent economic value will help to study the trade-off between such values and user privacy within the advertising system. This will require new proposals for complex machine learning techniques to enhance ads *targeting* as previous works found that the majority of received ads were not tailored to intended user-profiles [27, 44], which will ultimately help advertising systems to increase their revenues and enhance user experience in receiving relevant ads.

When introducing novel privacy-preserving mechanisms, a very basic step would be to combine various proposals, as described in Sect. 6, which will result in more robust and useful privacy solutions for various purposes: enhanced user *targeting*, invasive tracking behaviours, better adapting privacy-enhancing technologies, improved flexibility in regards to the changing economic aspects and *ethics* to ads *targeting*.

Another research direction would be to extend the analysis of privacy protection mechanisms to other players, such as advertisers, ad exchange, and publishers, with the aim of analysing and evaluating privacy policies and protection mechanisms that are claimed by these parties. This would help various entities in the advertising system to identify the flaws and further improve their working environment.

A further research direction would be to create smarter privacy protection tools on the user side, i.e. to create such tools as an essential component of mobile/browser-based platforms within the advertising ecosystem. To develop such tools where users effectively enforce various protection strategies, requires various important parameters of usability, flexibility, scalability, etc., to be considered to give users transparency and control over their private data.^34^

### Private billing mechanism

Billing for both *ad presentations* and *clicks* is an important component of online advertising system. As discussed in Appendix B, a private *billing* proposal is based on *Threshold BLS signature*, *Polynomial commitment*, and *Zero knowledge proof* (ZKP), which are based on PIR mechanisms and *Shamir secret sharing* scheme along with *Byzantine robustness*. The applicability of this private *billing* model can be verified in the online advertising system, which would require changes on both the user and ad system side. Furthermore, note that the this private *billing* mechanism, implemented via *polynomial commitment* and *zero-knowledge proof*, is highly resource-consuming process; henceforth, an alternative implementation with reduced processing time and query request size can be achieved via implementing together *billing* with PIR using *multi-secret sharing* scheme. In addition, to explore the effect of *multi-secret sharing* scheme in multiple-server PIR and hence comparative analysis to choose between the two variations of *single-secret* and *multi-secret sharing* system implementations. *Multi-secret sharing* scheme would help reduce the communication bandwidth and delays along with the processing time of query requests/responses

In addition, our *billing* mechanism for *ad presentations* and *clicks* presented in [8] is applicable only to single ad requests with no impact on privacy. However, having broader parameter values (simultaneously processing multiple ad requests) and utilising other PIR techniques, such as Hybrid-PIR [123] and Heterogeneous-PIR [207], can increase the efficiency of use of the processing time.

Furthermore, with the rise in popularity of Cryptocurrencies, many businesses and individuals have started investing in them, henceforth, the applicability of embedding the Cryptocurrency with the existing *billing* methods needs an investigation and development of new frameworks for coexisting the *billing* payments with the Cryptocurrency market. In addition, this would require techniques for purchasing, selling, and transferring Cryptocurrency among various parties, i.e. ad systems, *app* developers, publishers, advertisers, crypto-markets, and miners. Further analysis would require investigating the impact of such proposals on the current advertising business model with/without a significant effect.

An important research direction is to explore the implementation of private advertising systems in Blockchain networks since there are limited Blockchain-based advertising systems, e.g. [36, 41]. The [36] presents the design of a decentralised framework for *targeted* ads that enables private delivery of ads to users whose behavioural profiles accurately match the presented ads, defined by the advertising systems. This framework provides: a private *profiling* mechanism, privately requesting ads from the advertising system, the *billing* mechanisms for ads monetisation, uploading ads to the cloud system, various types of transactions to enable advertising operations in Blockchain-based network, and *access policy* over cloud system for accessing various resources (such as ads, mobile user profiles). However, its applicability in an actual environment is still questionable, in addition to, the coexistence of *ads-billing* mechanism with Cryptocurrency.

## Conclusion

In this paper, we provide a comprehensive overview of privacy issues and solutions in mobile-based targeted advertising systems. Considering the proposed and in some cases used (in the industry) privacy-preserving approaches, we have found that it is extremely difficult to provide user privacy in a way that enables greater user control of their private data and to simultaneously reduce the financial impact of introducing privacy mechanisms, without significantly changing the advertising ecosystems and their operations. To aid further development of privacy-enabled ad systems, we also identify open research issues that need to be solved in future work.

## Data Availability

This manuscript has no associated data.

## Appendix A: private information retrieval (PIR)

PIR [117, 118, 123, 164–166] is a multi-party cryptographic protocol where users can retrieve their desired items from a database without letting the database server know about the item(s) being retrieved. We used various variants of the PIR schemes [8] to enable secure communication within the existing advertising systems and a secure payment scheme for billing for ads being clicked or presented to the users. Our primary motivation for using PIR, compared to other solutions such as Oblivious Transfer [169, 170], is to enable lower communication and computation overheads.

Let a user wishes to privately retrieve ${\beta ^{th}}$ record(s) from the database *D* where is $r \times s$; *r* is total records in *D*, *s* is the size of each record, which may be divided into words of size *w*. For *multi-server* PIR, a scheme uses *l* database servers and has a privacy level of *t*; *k* is the number of servers that respond to the client’s query; among those, there are *v* Byzantine servers (i.e. malicious servers that respond incorrectly) and *h* honest servers that send a correct response to the client’s query. Following, we briefly discuss and compare various PIR schemes.

### Appendix A.1: computational PIR (CPIR)

The *single-server* PIR schemes, such as CPIR [116], rely on computational complexity (under the assumption that an adversary has limited resources) to ensure privacy against malicious adversaries. To privately retrieve the ${\beta \textrm{th}}$ record from *D*, a CPIR client creates a matrix ${M_\beta }$ by adding hard noise (based on large disturbance by replacing each diagonal term in ${M_\beta }$ by a random bit of $2^{40}$ words [116]) to the desired record and soft noise (based on small disturbance) to all the other records. The client assumes that the server cannot distinguish between the matrices with hard and soft noises. The server multiplies the query matrix ${M_\beta }$ to the database *D* that results in corresponding response *R*; the client removes the noise from *R* to derive the requested record ${\beta \textrm{th}}$.

### Appendix A.2: recursive CPIR (R-CPIR)

The CPIR mechanism is further improved in terms of communication costs [116] by recursively using the *single-server* CPIR where the database is split into a set of virtual small recordsets each considered as a virtual database. The query is hence calculated against part of the database during each recursion. The client recursively queries for the virtual records, each recursion results in a virtual database of smaller virtual records, until it determines a single (actual) record that is finally sent to the client.

### Appendix A.3: information theoretic PIR (IT-PIR)

The *multi-server* IT-PIR schemes [118–122] rely on multiple servers to guarantee privacy against colluding adversaries (that have unbounded processing power) and additionally provide *Byzantine robustness* against malicious servers.

To query a database for ${\beta \textrm{th}}$ record with protection against up to *t* colluding servers, the client first creates a vector ${e_\beta }$, with ‘1’ in the ${\beta \textrm{th}}$ position and ‘0’ elsewhere. The client then generates $\left( {l,t} \right)$ *Shamir secret shares* $v_1, v_2, \cdots , v_l$ for ${e_\beta }$. The shares (one each) are subsequently distributed to the servers. Each server *i* computes the response as ${R_i} = {v_i} \cdot D$, this is sent back to the client. The client reconstructs the requested ${\beta \textrm{th}}$ record of the database from these responses. The use of of *Shamir secret sharing* enables the recovery of the desired record from (only) $k \le l$ server responses [118], where $k > t$ (and $t < l$).

### Appendix A.4: hybrid-PIR (H-PIR)

The *multi-server* H-PIR scheme [123] combines *multi-server* IT-PIR [118] with the recursive nature of the *single-server* CPIR [116] to improve performance, by lowering the computation and communication costs.^35^ Let these two schemes be, respectively, represented by $\tau$ for IT-PIR and the $\gamma$ for the recursive CPIR protocol. If a client wants to retrieve ${\beta \textrm{th}}$ record, then the client must determine the index of virtual records containing the desired records at each step of the recursion until the recursive depth *d*. The client creates an IT-PIR $\tau$-query for the first index and sends it to each server. It then creates CPIR $\gamma$-query during each of the recursive steps and sends it to all the servers. Similarly, on the server-side at each recursive step; the server splits the database into virtual records each containing actual records, uses the $\tau$ server computation algorithm, and finally uses the $\gamma$ CPIR server computation algorithm. The last recursive step results in the record ${R_i}$, which is sent back to the client.

### Appendix A.5: comparison and applicability of various PIR techniques in ad systems

Following comparative analysis, based on literature work, would help the selection of various PIR schemes and their applicability within an advertising system. We note that various performance metrics relate to the size of the query along with the selection of a particular PIR scheme, e.g. the CPIR takes longer processing delays and highest bandwidth consumption compared to both the IT-PIR and H-PIR schemes. This is due to the computations involved in query encoding and due to the servers performing *matrix-by-matrix* computations instead of *vector-by-matrix*, as is used by the IT-PIR and H-PIR schemes [123], although the communication cost can be lowered down using the recursive version of the CPIR [116].

Furthermore, IT-PIR provides some other improvements, such as the *robustness*, which is its ability to retrieve correct records even if some of the servers do not respond or incorrectly/maliciously respond [122]. It is further evident [123] that both the *single-server* CPIR and the *multi-server* IT-PIR schemes, such as [118–121], respectively, make the assumptions of computationally bounded and that particular threshold of the servers are not colluding to discover the contents of a client’s query. Alternatively, the H-PIR [123], provides improved performance by combining *multi-server* IT-PIR with the recursive nature of *single-server* CPIR schemes, respectively, to improve the computation and communication costs.

A recent implementation, i.e. Heterogeneous PIR [207], enables *multi-server* PIR protocols (implemented using multi-secret sharing algorithm, compatible with *Percy++*^36^ PIR library) over non-uniform servers (in a heterogeneous environment where servers are equipped with diverse resources e.g. computational capabilities) that impose different computation and communication overheads. This implementation makes it possible to run PIR over a range of different applications e.g. various resources (ad’s contents such as JPEG, JavaScript files) present on CDN in distributed environments. Furthermore, this implementation has tested and compared its performance with Goldberg’s [118] implementation with different settings, e.g. for different database sizes, numbers of queries, and various degrees of heterogeneity. This implementation achieves a trade-off between computation and communication overheads in heterogeneous server implementation by adjusting various parameters.

## Appendix B: building blocks for enabling PIR and private billing

This section introduces various building blocks for enabling PIR techniques, i.e. *Shamir secret sharing* and *Byzantine robustness*. It further discusses various techniques that are used for private *billing*, i.e. *Threshold BLS signature*, *Polynomial commitment*, and *Zero-knowledge proof* (ZKP).

### Appendix B.1: Shamir secret sharing

The *Shamir secret sharing* [208] scheme divides a *secret* $\sigma$ into parts, giving each participant e.g. *l* servers a unique part where some or all of the parts are needed in order to reconstruct the *secret*. If the *secret* is found incorrect, then it can be handled through error-correcting codes, such as the one discussed in [209]. Let the $\sigma$ be an element of some finite field *F*, then the *Shamir scheme* works as follows: a client selects an *l* distinct nonzero elements ${\alpha _1},{\alpha _2}, \cdots ,{\alpha _l} \in F$ and selects *t* elements ${a_1},{a_2}, \cdots ,{a_t}{ \in _R}F$ (the ${ \in _R}$ means uniformly at random). A polynomial $f\left( x \right) = \sigma + {a_1}x + {a_2}{x^2} + \cdots + {a_t}{x^t}$ is constructed and gives the share $\left( {{\alpha _i},f\left( {{\alpha _i}} \right) } \right) \in F \times F$ to the server *i* for $1 \le i \le l$. Now any $t + 1$ or more servers can use Lagrange interpolation [122] to reconstruct the polynomial *f* and, similarly, obtains $\sigma$ by evaluating $f\left( 0 \right)$.

### Appendix B.2: Byzantine robustness

The problem of *Byzantine* failure allows a server to continue its operation but it incorrectly responds. The *Byzantine* failure may include corrupting messages, forging messages, or sending conflicting messages through malice or errors. In order to ensure the responses’ integrity in a *single-server*, such as PIR-Tor [210], the server can provide a *cryptographic signature* on each database’s block. However, in a *multi-server* PIR environment, the main aim of the *Byzantine robustness* is to ensure that the protocol still works correctly when some of the servers fail to evaluate responses or incorrectly/maliciously respond. The client at the same time might also be interested in figuring out which servers have sent incorrect responses so that they can be avoided in the future.

The *Byzantine robustness* for PIR was first considered by Beimel and Stahl [211, 212]; the scheme called the *t*-private *v*-*Byzantine robust* *k*-out-of-*l* PIR. The authors take the *l*-server IT-PIR setting where *k* servers correctly respond, *v* servers provide incorrect responses, and the system can sustain up to *t* colluding servers to privately retrieve the client’s query. Furthermore, they suggest the *unique decoding* where the protocol always outputs a correct unique block under the conditions $v \le t \le {k/3}$.

The [118] uses the *list decoding*, which is an substitute of unique decodingscheme that is used for error-correcting codes when the system experiences large error rates, and demonstrates that the privacy level can be substantially increased up to $0< t < k$ and the protocol can tolerate up to $k - \left\lfloor {\sqrt{kt} } \right\rfloor - 1$ *Byzantine* servers. Alternatively, the *list decoding* can also be converted to *unique decoding* [213] at the cost of slightly increasing the database size [122].

Following schemes are the essential building blocks for enabling private *billing* along with evaluating the PIR techniques for privately retrieving ads from the ad database.

### Appendix B.3: threshold BLS signature

The *Boneh-Lynn-Shacham* (BLS) [214] is a ‘short’ *signature* verification scheme that allows a user to verify that the signer is authentic. The signer’s private key is a random integer $x \in {Z_q}$ whereas the corresponding public key is $\left( {{\hat{g}}, {\hat{g}}^{x}} \right)$ (${\hat{g}}$ is a generator of ${{\mathbb {G}}_2}$). The procedure for *signature* verification is as follows: The *signature* is computed, using *x* key and *m* message, via $\sigma = {h_x}$ where the $h = hash(m)$ is the cryptographic hash of *m*; the verification equation is $$e(\sigma ,{\hat{g}})\begin{array}{*{20}{c}} ?\\ = \end{array}e(h, {\hat{g}}^{x})$$, which results in true/false. To fit into scenario of multiple PIR servers; a $\left( {k,l} \right)$-threshold variant of *BLS signature* can be used where signing keys are the evaluations of a polynomial of degree $\left( k - l \right)$ and the master *secret* is the constant term. Similarly, the reconstruction process can be done using Lagrange interpolation. The $\left( k - l \right)$ threshold *BLS signature* partly provides *robustness* against the *Byzantine* signers since the verifier can independently verify the *signature* using the public key.

### Appendix B.4: polynomial commitment

A *polynomial commitment* [215] scheme allows committers to formulate a constant-sized *commitments* to polynomials that s(he) can commit so that it can be used by a verifier to confirm the stated evaluations of the committed polynomial [216], while keeping secret additional information regarding the committed value(s). An example of the *Polynomial commitment* constructions in [215] provides unconditional hiding when a *commitment* is revealed to a majority of $t - 1$ evaluations (i.e. $t - 1$ servers for a degree-*t* polynomial). Additionally, it provides computational hiding under the discrete *log*(*DL*) if polynomial *commitment* is opened to at least *t* evaluations. As presented in [215], *commitment* to a polynomial $f\left( x \right) = {a_t}{x^t} + \cdots + {a_1}z + {a_0}$ has the form ${{{\mathscr {C}}}_f} = {\left( {{g^{{\alpha ^t}}}} \right) ^{{a_t}}} \cdots {\left( {{g^\alpha }} \right) ^{{a_1}}}{g^{{a_0}}} = {g^{f\left( \alpha \right) }}$ where $\alpha$ is *secret*, $g \in {{\mathbb {G}}_1}$ is a generator whose discrete log with respect to *g* is unknown, including all the bases are part of the *commitment* scheme’s *public key*. The verifier, on the other side, can confirm that the claimed evaluations is true by checking if $Ver\left( {{{{\mathscr {C}}}_f},r,f\left( r \right) ,w} \right) = \left[ {e\left( {{{{\mathscr {C}}}_f},{{\hat{g}}}} \right) \mathop = \limits ^? e\left( {w,{{{{\hat{g}}^{\alpha }}} / {{{\hat{g}}^{r}}}}} \right) .e{{\left( {g,{{\hat{g}}}} \right) }^{f\left( r \right) }}} \right]$ is true, here the *commitment* *w* is called the *witness*; detailed discussion can be found in [215].

### Appendix B.5: zero-knowledge proof (ZKP)

The *zero knowledge proof* is an interactive protocol that allows the *prover* to prove to the *verifier* that it privately holds a given statement while revealing no additional information. There are several *ZKPs*, such as range proof to prove that a committed value is non-negative [171], the proof of knowledge of a committed value [172], knowledge proof of a discrete log representation of a number [173], and proof that a *commitment* opens to multiple *commitments* [174]. Besides, there are several batch proof techniques, such as [217, 218] to achieve verification of a basic operation like modular exponentiation in some groups, which significantly reduces the computation time.

## Appendix C: k-anonymity

*k-anonymity* was introduced in [112, 219] and its enforcement via generalisation and suppression was suggested in [113]. *k-anonymity* examines the re-identification attack, which aims to release a private version of the data (i.e. structured data e.g. data holders of banks or hospitals etc.) that the attacker cannot re-identify the actual data while the anonymised data remain useful. Let $RT\left( {{A_1}, \ldots ,{A_n}} \right)$ be a set of structured data organised in rows and columns, a population of entities *U*, with a finite set of attributes of $RT$as $\left( {{A_1}, \ldots ,{A_n}} \right)$ with at least one attribute identified as ‘*key attribute*’ that can be considered as *quasi-identifier*.^37^ A *quasi-identifier* of *RT*, represented as ${Q_{RT}}$, is a set of attributes $\left( {{A_1}, \ldots ,{A_j}} \right) \subseteq \left( {{A_1}, \ldots ,{A_n}} \right)$, where $\exists {p_i} \subset U$ such that ${f_g}\left( {{f_c}\left( {{p_i}} \right) \left[ {{Q_{RT}}} \right] } \right) = {p_i}$; ${f_c}:U \rightarrow RT$ and ${f_g}:RT \rightarrow U'$, $U \subseteq U'$.

*k-anonymity* for *RT* is achieved if the sequence of values in $RT\left[ {{Q_{RT}}} \right]$ repeats with a maximum of *k* occurrences, i.e. ${Q_{RT}} = \left( {{A_1}, \ldots ,{A_j}} \right)$ is the *quasi-identifier* associated with *RT*, where ${A_1}, \ldots ,{A_j} \subseteq {A_1}, \ldots ,{A_n}$ and *RT* fulfils the requirements of *k-anonymity*. Subsequently, each sequence of values in $RT\left[ {{A_x}} \right]$ repeats with a maximum of *k* occurrences in $RT\left[ {{Q_{RT}}} \right]$ for $x = i, \ldots ,j$. The *RT* satisfies the *k-anonymity* is released. The combination of any set of attributes of released data *RT* and external sources on which ${{Q_{PT}}}$ (*PT* is the private table) is based, cannot be linked that eventually guarantees the privacy of released data. A comprehensive example is given in [112].

## Footnotes

1 https://www.statista.com/statistics/376260/global-ad-spend-distribution-by-medium/ (Accessed: Nov, 2022).

2 Analytics is the systematic computational analysis of data or statistics for deeper understanding of consumer requirements. E.g. Google Analytics https://analytics.google.com, Flurry Analytics https://www.flurry.com/analytics/. (Accessed: Nov, 2022).

3 https://www.adviso.ca/en/blog/tech-en/cookie-apocalypse/ (Accessed: Nov, 2022).

4 DMP is a unified and centralised technology platform used for collecting, organising, and activating large sets of data from disparate sources. DSP allows for advertisers to buy impressions across several different publisher sites, all targeted to specific users and based on key online behaviours and identifiers. See https://www.lotame.com/dmp-vs-dsp/ for discussion over DMP and DSP.

5 https://junction.cj.com/article/button-weighs-in-what-does-apples-idfa-opt-in-overhaul-mean-for-affiliate (Accessed: Nov, 2022).

6 https://www.theiet.org/publishing/inspec/ (Accessed: Nov, 2022).

7 https://dblp.org/ (Accessed: Nov, 2022).

8 https://dl.acm.org/ (Accessed: Nov, 2022).

9 https://ieeexplore.ieee.org/Xplore/home.jsp (Accessed: Nov, 2022).

10 Google AdMob profile is accessible through the *Google Settings* system *app* on Android devices, accessible through Google Settings $\rightarrow$ Ads $\rightarrow$ Ads by Google $\rightarrow$ Ads Settings.

11 Google profile interests are listed in https://adssettings.google.com/authenticated?hl=en, under the ‘How your ads are personalised’. Google Dashboard shows a list of Google services: https://myaccount.google.com/dashboard?hl=en (Accessed: Nov, 2022).

12 Demographic Targeting https://support.google.com/google-ads/answer/2580383?hl=en (Accessed: Nov, 2022).

13 https://myactivity.google.com/myactivity?otzr=1 (Accessed: Nov, 2022).

14 https://support.google.com/google-ads/answer/6308 (Accessed: Nov, 2022).

15 Google’s Connected Home Devices and Services: https://support.google.com/googlenest/answer/9327662?p=connected-devices &visit_id=637357664880642401-2675773861 &rd=1 (Accessed: Nov, 2022).

16 Sensors in Google Nest devices: https://support.google.com/googlenest/answer/9330256?hl=en (Accessed: Nov, 2022).

17 https://takeout.google.com/ (Accessed: Nov, 2022).

18 *Conversion tracking* is specifically used by Google that is an action a customer takes on the website that has value to the business, such as a purchase, a sign-up, or a view of a key page [34].

19 E.g. by modifying Google ads preferences: https://adssettings.google.com/authenticated?hl=en (Accessed: Nov, 2022).

20 https://www.osec.doc.gov/opog/privacy/PII_BII.html (Accessed: Nov, 2022).

21 http://au.pcmag.com/mobile-operating-system/31341/opinion/apple-ios-9-ad-blocking-explained-and-why-its-a-ba (Accessed: Nov, 2022).

22 My Google Activity: https://myactivity.google.com/myactivity?otzr=1 (Accessed: Nov, 2022).

23 Kaggle Dataset: https://www.kaggle.com/datasets, Dataset Search: https://datasetsearch.research.google.com/ (Accessed: Nov, 2022).

24 Access from Google Settings $\rightarrow$ Ads $\rightarrow$ Ads by Google $\rightarrow$ Ads Settings. It claims that Google’s ad network shows ads on 2+million non-Google websites and *apps*.

25 https://microsites.nielsen.com/daas-partners/partner/exelate/ (Accessed: Nov, 2022).

26 https://www.oracle.com/corporate/acquisitions/bluekai/ (Accessed: Nov, 2022).

27 https://www.addthis.com/ (Accessed: Nov, 2022).

28 Every Android *app* contains the *manifest* file that describes essential information about app, such as, *app ID*, *app name*, *permission to use device resources used by an app e.g. contacts, camera, list of installed apps etc.*, *hardware and software features the app requires* etc. https://developer.android.com/guide/topics/manifest/manifest-intro (Accessed: Nov, 2022).

29 https://optout.aboutads.info/?c=2 &lang=EN (Accessed: Nov, 2022).

30 Google Privacy & Terms: https://policies.google.com/technologies/anonymization?hl=en-US (Accessed: Nov, 2022).

31 Anonymizing data for data protection policies: https://www.ibm.com/docs/en/iis/11.7?topic=rules-anonymizing-data (Accessed: Nov, 2022).

32 A C++ implementation of *differential privacy* can be found at https://github.com/google/differential-privacy (Accessed: Nov, 2022).

33 A publicly available resource to see how your community is moving around differently due to COVID-19: http://google.com/covid19/mobility (Accessed: Nov, 2022).

34 It [202] proposes a DNT field in the HTTP header that requests a web application to either disable the tracking (where it is automatically set) or cross-site the user tracking of an individual user.

## References

[1] GreensMedia: 45 digital and targeted advertising statistics. https://www.grenismedia.com/blog/45-digital-and-targeted-advertising-statistics/ (2022)

[2] Buildfire: Number of mobile app downloads worldwide. https://www.statista.com/statistics/271644/worldwide-free-and-paid-mobile-app-store-downloads/ (2022)

[3] Grace, M. C., Zhou, W., Jiang, X., Sadeghi, A.-R.: Unsafe exposure analysis of mobile in-app advertisements. Proceedings of the fifth ACM conference on Security and Privacy in Wireless and Mobile Networks, pp. 101–112 (2012)

[4] Book, T., Wallach, D. S.: A case of collusion: a study of the interface between ad libraries and their apps. Proceedings of the Third ACM workshop on Security and privacy in smartphones & mobile devices, pp. 79–86 (2013)

[5] Chaabane, A., Acs, G., Kaafar, M. A.: You are what you like! information leakage through users’ interests. Proceedings of the 19th Annual Network & Distributed System Security Symposium (NDSS) (2012)

[6] Castelluccia, C., Kaafar, M.-A., Tran, M.-D.: “Betrayed by your ads!,” Springer, Privacy Enhancing Technologies (PETs), pp. 1–17 (2012)

[7] Estrada-Jiménez J, Parra-Arnau J, Rodríguez-Hoyos A, Forné J (2017). Online advertising: analysis of privacy threats and protection approaches. Comput. Commun..

[8] Ullah, I., Sarwar, B. G., Boreli, R., Kanhere, S. S., Katzenbeisser, S., Hollick, M.: Enabling privacy preserving mobile advertising via private information retrieval. 2017 IEEE 42nd Conference on Local Computer Networks (LCN), pp. 347–355 (2017)

[9] Ullah I, Boreli R, Kanhere SS, Chawla S, Ahanger TA, Tariq U (2020). Protecting private attributes in app based mobile user profiling. IEEE Access.

[10] Chen, T., Ullah, I., Kaafar, M. A., Boreli, R.: Information leakage through mobile analytics service. ACM HotMobile 15th International workshop on mobile computing systems and applications, (2014)

[11] Mamais, S.: Privacy-preserving and fraud-resistant targeted advertising for mobile devices. PhD thesis, Cardiff University, https://orca.cardiff.ac.uk/id/eprint/125897/1/2019mamaisssphd.pdf (2019)

[12] Liu Y, Simpson AA (2016). Privacy-preserving targeted mobile advertising: requirements, design and a prototype implementation. Softw. Pract. Exp..

[13] Wang Y, Genc E, Peng G (2020). Aiming the mobile targets in a cross-cultural context: effects of trust, privacy concerns, and attitude. Int. J. Hum. Comput. Interact..

[14] CNET: Facebook vs. apple: here’s what you need to know about their privacy feud. https://www.cnet.com/news/facebook-vs-apple-heres-what-you-need-to-know-about-their-privacy-feud/ (2022)

[15] Consulting, I.: EU general data protection regulation (GDPR). https://gdpr-info.eu/ (2022)

[16] Government, A.: The privacy act in Australia: federal register of legislation. https://www.legislation.gov.au/Series/C2004A03712 (2022)

[17] Bonta, R.: California consumer privacy act (CCPA). https://www.oag.ca.gov/privacy/ccpa (2022)

[18] Toch E, Wang Y, Cranor LF (2012). Personalization and privacy: a survey of privacy risks and remedies in personalization-based systems. User Model. User Adapt. Interact..

[19] Kaaniche N, Laurent M, Belguith S (2020). Privacy enhancing technologies for solving the privacy-personalization paradox: taxonomy and survey. J. Netw. Comput. Appl..

[20] Boerman SC, Kruikemeier S, Zuiderveen Borgesius FJ (2017). Online behavioral advertising: a literature review and research agenda. J. Advert..

[21] Webster, J., Watson, R. T.: Analyzing the past to prepare for the future: Writing a literature review, MIS quarterly, pp. xiii–xxiii (2002)

[22] Okoli C (2015). A guide to conducting a standalone systematic literature review. Commun. Assoc. Inf. Syst..

[23] Leontiadis, I., Efstratiou, C., Picone, M., Mascolo, C.: Don’t kill my ads!: balancing privacy in an ad-supported mobile application market. Proceedings of the ACM Twelfth workshop on mobile computing systems & applications, p. 2 (2012)

[24] Vallina-Rodriguez, N., Shah, J., Finamore, A., Grunenberger, Y., Papagiannaki, K., Haddadi, H., Crowcroft, J.: Breaking for commercials: characterizing mobile advertising. Proceedings of the 2012 ACM conference on internet measurement conference, pp. 343–356 (2012)

[25] Han, S., Jung, J., Wetherall, D.: A study of third-party tracking by mobile apps in the wild, Univ. Washington, Tech. Rep. UW-CSE-12-03. http://dada.cs.washington.edu/research/tr/2012/03/UW-CSE-12-03-01.PDF, vol. 1 (2012)

[26] Flurry advertisers, publishers, and analytics http://www.flurry.com (2022)

[27] Ullah, I., Boreli, R., Kaafar, M. A., Kanhere, S. S.: Characterising user targeting for in-app mobile ads. 2014 IEEE Conference on computer communications workshops (INFOCOM WKSHPS), pp. 547–552, (2014)

[28] Mobile advertising market size, share & industry analysis, forecast 2019-2026. https://www.fortunebusinessinsights.com/mobile-advertising-market-102496 (2022)

[29] Ng V, Ho MK (2002). An intelligent agent for web advertisements. World Sci. Int. J. Found. Comput. Sci..

[30] Thawani, A., Gopalan, S., Sridhar, V.: Event driven semantics based ad selection, multimedia and expo, 2004. 2004 IEEE International Conference on ICME’04. vol. 3, pp. 1875–1878, (2004)

[31] Yan, J., Liu, N., Wang, G., Zhang, W., Jiang, Y., Chen, Z.: How much can behavioral targeting help online advertising? Proceedings of the ACM 18th international conference on World wide web, pp. 261–270, (2009)

[32] Jaworska, J., Sydow, M: Behavioural targeting in on-line advertising: an empirical study. In: International conference on web information systems engineering, pp. 62–76. Springer, Heidelberg (2008)

[33] Shin, J., Yu, J.: Targeted advertising: how do consumers make inferences? School of Management, Yale University. https://tinyurl.com/y582epra (2019)

[34] Tracking, C.: Understanding conversion tracking, Google Support. http://support.google.com/adwords/bin/answer.py?hl=en &answer=1722022 (2022)

[35] Ullah I, Binbusayyis A (2022). Joint optimization of privacy and cost of in-app mobile user profiling and targeted ads. IEEE Access.

[36] Ullah, I., Kanhere, S. S., Boreli, R.: Privacy-preserving targeted mobile advertising: a blockchain-based framework for mobile ads. *arXiv preprint* arXiv:2008.10479 (2020)

[37] Guha, S., Cheng, B., Reznichenko, A., Haddadi, H., Francis, P.: Privad: Rearchitecting online advertising for privacy. Proceedings of Hot Topics in Networking (HotNets) (2009)

[38] Toubiana, V., Narayanan, A., Boneh, D., Nissenbaum, H., Barocas, S.: Adnostic: Privacy preserving targeted advertising. Proceedings Network and Distributed System Symposium (2010)

[39] Rafieian O, Yoganarasimhan H (2021). Targeting and privacy in mobile advertising. Market. Sci..

[40] Ullah, I., Boreli, R., Kanhere, S. S., Chawla, S.: Profileguard: Privacy preserving obfuscation for mobile user profiles. Proceedings of the 13th ACM Workshop on Privacy in the Electronic Society, pp. 83–92 (2014)

[41] Gu, Y., Gui, X., Xu, P., Gui, R., Zhao, Y., Liu, W.: A secure and targeted mobile coupon delivery scheme using blockchain. International Conference on Algorithms and Architectures for Parallel Processing, pp. 538–548 (2018)

[42] Trzcinski T (2011). Analyse, target & advertise privacy in mobile ads.

[43] Khan, A. J., Jayarajah, K., Han, D., Misra, A., Balan, R., Seshan, S.: Cameo: a middleware for mobile advertisement delivery. Proceeding of the ACM 11th annual international conference on Mobile systems, applications, and services, pp. 125–138 (2013)

[44] Nath, S.: Madscope: characterizing mobile in-app targeted ads. Proceedings of the 13th ACM annual international conference on mobile systems, applications, and services, pp. 59–73 (2015)

[45] Haddadi, H., Hui, P., Brown, I.: Mobiad: private and scalable mobile advertising. Proceedings of the fifth ACM international workshop on Mobility in the evolving internet architecture, pp. 33–38 (2010)

[46] Balebako, R., Leon, P., Shay, R., Ur, B., Wang, Y., Cranor, L.: Measuring the effectiveness of privacy tools for limiting behavioral advertising. Web 2.0 Security and Privacy Workshop, (2012)

[47] Wills, C. E., Tatar, C.: Understanding what they do with what they know. Proceedings of the 2012 ACM Workshop on Privacy in the Electronic Society, pp. 13–18 (2012)

[48] Goldfarb A, Tucker C (2011). Online display advertising: targeting and obtrusiveness. Market. Sci..

[49] Farahat, A., Bailey, M. C.: How effective is targeted advertising? Proceedings of the ACM 21st international conference on World Wide Web, pp. 111–120 (2012)

[50] Evans DS (2009). The online advertising industry: economics, evolution, and privacy. J. Econ. Perspect..

[51] Barford, P., Canadi, I., Krushevskaja, D., Ma, Q., Muthukrishnan, S.: Adscape: harvesting and analyzing online display ads. Proceedings of the ACM 23rd international conference on World wide web, pp. 597–608 (2014)

[52] Mohan, P., Nath, S., Riva, O.: Prefetching mobile ads: can advertising systems afford it? Proceedings of the 8th ACM European Conference on Computer Systems, pp. 267–280 (2013)

[53] Xu, Q., Erman, J., Gerber, A., Mao, Z., Pang, J., Venkataraman, S.: Identifying diverse usage behaviors of smartphone apps. Proceedings of the ACM SIGCOMM conference on Internet measurement conference, pp. 329–344 (2011)

[54] Lee, S.-W., Park, J.-S., Lee, H.-S., Kim, M.-S.: A study on smart-phone traffic analysis. IEEE Network Operations and Management Symposium (APNOMS), 2011 13th Asia-Pacific, pp. 1–7 (2011)

[55] Zhang L, Gupta D, Mohapatra P (2012). How expensive are free smartphone apps?. ACM SIGMOBILE Mob. Comput. Commun. Rev..

[56] Pathak, A., Hu, Y. C., Zhang, M.: Where is the energy spent inside my app?: fine grained energy accounting on smartphones with eprof. Proceedings of the 7th ACM european conference on Computer Systems, pp. 29–42 (2012)

[57] Pathak, A., Hu, Y. C., Zhang, M., Bahl, P., Wang, Y.-M.: Fine-grained power modeling for smartphones using system call tracing. Proceedings of the sixth ACM conference on Computer systems, pp. 153–168 (2011)

[58] Qian, F., Wang, Z., Gerber, A., Mao, Z., Sen, S., Spatscheck, O.: Profiling resource usage for mobile applications: a cross-layer approach. Proceedings of the 9th ACM international conference on Mobile systems, applications, and services, pp. 321–334 (2011)

[59] Razaghpanah, A., Nithyanand, R., Vallina-Rodriguez, N., Sundaresan, S., Allman, M., Kreibich, C., Gill, P.: Apps, trackers, privacy, and regulators: a global study of the mobile tracking ecosystem (2018)

[60] Elsabagh, M., Johnson, R., Stavrou, A., Zuo, C., Zhao, Q., Lin, Z.: FIRMSCOPE: Automatic uncovering of privilege-escalation vulnerabilities in pre-installed apps in android firmware. In: 29th USENIX Security Symposium (USENIX Security 20) (2020)

[61] Ren, J., Rao, A., Lindorfer, M., Legout, A., Choffnes, D.: Recon: revealing and controlling pii leaks in mobile network traffic. Proceedings of the 14th Annual International Conference on Mobile Systems, Applications, and Services, pp. 361–374 (2016)

[62] Verderame, L., Caputo, D., Romdhana, A., Merlo, A.: On the (un) reliability of privacy policies in android apps. 2020 IEEE International Joint Conference on Neural Networks (IJCNN), pp. 1–9 (2020)

[63] Lécuyer, M., Ducoffe, G., Lan, F., Papancea, A., Petsios, T., Spahn, R., Chaintreau, A., Geambasu, R.: Xray: Enhancing the web’s transparency with differential correlation. 23rd USENIX Security Symposium (USENIX Security 14). San Diego, CA (2014)

[64] Gandhi M, Jakobsson M, Ratkiewicz J (2006). Badvertisements: stealthy click-fraud with unwitting accessories. J. Digit. Forensic Pract..

[65] Guha, S., Cheng, B., Francis, P.: Challenges in measuring online advertising systems. Proceedings of the 10th ACM SIGCOMM conference on Internet measurement, pp. 81–87 (2010)

[66] Solove, D. J.: Understanding privacy. Harvard University Press, https://papers.ssrn.com/sol3/Delivery.cfm/SSRN_ID1127888_code254274.pdf?abstractid=1127888 &mirid=1 (2008)

[67] Datta, A., Tschantz, M. C., Datta, A.: Automated experiments on ad privacy settings: A tale of opacity, choice, and discrimination. *arXiv preprint* arXiv:1408.6491 (2014)

[68] Rao, A., Schaub, F., Sadeh Koniecpol, N.: What do they know about me? contents and concerns of online behavioral profiles (CMU-CyLab-14-011). Carnegie Mellon University (2014)

[69] Book, T., Wallach, D. S.: An empirical study of mobile ad targeting. * arXiv preprint* arXiv:1502.06577 (2015)

[70] Stevens, R., Gibler, C., Crussell, J., Erickson, J., Chen, H.: Investigating user privacy in android ad libraries. Workshop on Mobile Security Technologies (MoST) (2012)

[71] Liu X, Liu J, Zhu S, Wang W, Zhang X (2019). Privacy risk analysis and mitigation of analytics libraries in the android ecosystem. IEEE Trans. Mob. Comput..

[72] Pearce, P., Felt, A. P., Nunez, G., Wagner, D.: Addroid: Privilege separation for applications and advertisers in android. Proceedings of the 7th ACM Symposium on Information, Computer and Communications Security, pp. 71–72 (2012)

[73] Shekhar, S., Dietz, M., Wallach, D. S.: Adsplit: separating smartphone advertising from applications. USENIX Security Symposium, pp. 553–567 (2012)

[74] Book, T., Pridgen, A., Wallach, D. S.: Longitudinal analysis of android ad library permissions. *arXiv preprint* arXiv:1303.0857 (2013)

[75] Aggarwal, G., Muthukrishnan, S., Pál, D., Pál, M.: General auction mechanism for search advertising. Proceedings of the 18th ACM international conference on World Wide Web (WWW), pp. 241–250 (2009)

[76] Guha, S., Reznichenko, A., Tang, K., Haddadi, H., Francis, P.: Serving ads from localhost for performance, privacy, and profit. HotNets (2009)

[77] Krishnamurthy, B., Wills, C. E.: On the leakage of personally identifiable information via online social networks. Proceedings of the 2nd ACM workshop on Online social networks, pp. 7–12 (2009)

[78] Krishnamurthy, B., Wills, C. E.: Privacy leakage in mobile online social networks,” USENIX Association. Proceedings of the 3rd conference on online social networks, p. 4 (2010)

[79] Metwally, A., Agrawal, D., El Abbadi, A.: Detectives: detecting coalition hit inflation attacks in advertising networks streams. Proceedings of the 16th ACM international conference on World Wide Web, pp. 241–250 (2007)

[80] Wang, Y., Burgener, D., Kuzmanovic, A., Maciá-Fernández, G.: Understanding the network and user-targeting properties of web advertising networks. 2011 31st International Conference on IEEE, Distributed Computing Systems (ICDCS), pp. 613–622 (2011)

[81] Schwartz HA, Eichstaedt JC, Kern ML, Dziurzynski L, Ramones SM, Agrawal M, Shah A, Kosinski M, Stillwell D, Seligman ME (2013). Personality, gender, and age in the language of social media: The open-vocabulary approach. Public Library of Science. PLoS One.

[82] Kosinski M, Stillwell D, Graepel T (2013). Private traits and attributes are predictable from digital records of human behavior. Proc. Natl. Acad. Sci..

[83] Goel, S., Hofman, J. M., Sirer, M. I.: Who does what on the web: a large-scale study of browsing behavior. International Conference on Web and Social Media (ICWSM). (2012)

[84] Hu, J., Zeng, H.-J., Li, H., Niu, C., Chen, Z.: Demographic prediction based on user’s browsing behavior. Proceedings of the 16th ACM international conference on World Wide Web, pp. 151–160 (2007)

[85] Schler, J., Koppel, M., Argamon, S., Pennebaker, J.W.: Effects of age and gender on blogging. AAAI: Computational Approaches to Analyzing Weblogs, pp. 199–205 (2006)

[86] Otterbacher, J.: Inferring gender of movie reviewers: exploiting writing style, content and metadata. Proceedings of the 19th ACM international conference on Information and knowledge management, pp. 369–378 (2010)

[87] Mukherjee, A., Liu, B.: Improving gender classification of blog authors. Proceedings of the 2010 conference on Empirical Methods in natural Language Processing, pp. 207–217 (2010)

[88] Bi, B., Shokouhi, M., Kosinski, M., Graepel, T.: Inferring the demographics of search users: social data meets search queries. 22nd International conference on World Wide Web (WWW), pp. 131–140 (2013)

[89] Ying, J. J.-C., Chang, Y.-J., Huang, C.-M., Tseng, V. S.: Demographic prediction based on users mobile behaviors. Mobile data challenge, (2012)

[90] Pennebaker, J.W., Francis, M.E., Booth, R.J.: Linguistic inquiry and word count: Liwc 2001. Mahway: Lawrence Erlbaum Associates, **71** (2001)

[91] Zheleva, E., Getoor, L.: To join or not to join: the illusion of privacy in social networks with mixed public and private user profiles. Proceedings of the 18th ACM international conference on World Wide Web (WWW), pp. 531–540 (2009)

[92] He, J., Chu, W. W. , Liu, Z. V.: Inferring privacy information from social networks. Intelligence and security informatics, Springer, pp. 154–165 (2006)

[93] Mislove, A., Viswanath, B., Gummadi, K. P., Druschel, P.: You are who you know: inferring user profiles in online social networks. Proceedings of the third ACM international conference on Web search and data mining, pp. 251–260 (2010)

[94] Ryu, E., Rong, Y., Li, J., Machanavajjhala, A.: curso: protect yourself from curse of attribute inference: a social network privacy-analyzer. Proceedings of the ACM SIGMOD workshop on databases and social networks, pp. 13–18 (2013)

[95] Enck W, Gilbert P, Han S, Tendulkar V, Chun B-G, Cox LP, Jung J, McDaniel P, Sheth AN (2014). Taintdroid: an information-flow tracking system for realtime privacy monitoring on smartphones. ACM Trans. Comput. Syst. (TOCS).

[96] Ongtang M, McLaughlin S, Enck W, McDaniel P (2012). Semantically rich application-centric security in android. Secur. Commun. Netw..

[97] Frik, A., Haviland, A., Acquisti, A.: The impact of ad-blockers on product search and purchase behavior: a lab experiment. 29th USENIX Security Symposium (USENIX Security 20) (2020)

[98] Shuba A, Markopoulou A (2020). Nomoats: towards automatic detection of mobile tracking. Proc. Priv. Enhancing Technol. (PETs).

[99] Iqbal, U., Snyder, P., Zhu, S., Livshits, B., Qian, Z., Shafiq, Z.: Adgraph: A graph-based approach to ad and tracker blocking. Proceedings of IEEE symposium on security and privacy (2020)

[100] Felt, A. P., Ha, E., Egelman, S., Haney, A., Chin, E., Wagner, D.: Android permissions: user attention, comprehension, and behavior. Proceedings of the eighth symposium on usable privacy and security, pp. 1–14 (2012)

[101] Felt, A. P., Wang, H. J., Moshchuk, A., Hanna, S., Chin, E.: Permission re-delegation: attacks and defenses. Proceedings of 20th USENIX Security Symposium (2011)

[102] Felt, A. P., Chin, E., Hanna, S., Song, D., Wagner, D.: Android permissions demystified. Proceedings of the 18th ACM conference on Computer and communications security, pp. 627–638 (2011)

[103] Chan, P. P., Hui, L. C., Yiu, S.-M.: Droidchecker: analyzing android applications for capability leak. Proceedings of the fifth ACM conference on Security and Privacy in Wireless and Mobile Networks, pp. 125–136 (2012)

[104] Enck, W., Ongtang, M., McDaniel, P.: On lightweight mobile phone application certification. Proceedings of the 16th ACM conference on Computer and communications security, pp. 235–245 (2009)

[105] Beresford, A. R., Rice, A., Skehin, N., Sohan, R.: Mockdroid: trading privacy for application functionality on smartphones. Proceedings of the 12th ACM Workshop on Mobile Computing Systems and Applications, pp. 49–54 (2011)

[106] Hornyack, P., Han, S., Jung, J., Schechter, S., Wetherall, D.: These aren’t the droids you’re looking for: retrofitting android to protect data from imperious applications. Proceedings of the 18th ACM conference on computer and communications security, pp. 639–652 (2011)

[107] Golle, P., Partridge, K.: On the anonymity of home/work location pairs. International Conference on Pervasive Computing, Springer, pp. 390–397 (2009)

[108] Zang, H., Bolot, J.: Anonymization of location data does not work: a large-scale measurement study. Proceedings of the 17th annual international conference on Mobile computing and networking, pp. 145–156 (2011)

[109] Mohammed, N., Fung, B. C., Debbabi, M.: Walking in the crowd: anonymizing trajectory data for pattern analysis. Proceedings of the 18th ACM conference on Information and knowledge management, pp. 1441–1444 (2009)

[110] Bonchi F, Lakshmanan LV, Wang H (2011). Trajectory anonymity in publishing personal mobility data. ACM Sigkdd Explor. Newsl..

[111] Shokri, R., Theodorakopoulos, G., Danezis, G., Hubaux, J.-P., Le Boudec, J.-Y.: Quantifying location privacy: the case of sporadic location exposure. International Symposium on Privacy Enhancing Technologies Symposium, Springer, pp. 57–76 (2011)

[112] Samarati P (2001). Protecting respondents identities in microdata release. IEEE Trans. Knowl. Data Eng..

[113] Sweeney L (2002). k-Anonymity: a model for protecting privacy. Int. J. Uncertain. Fuzziness Knowl. Based Syst..

[114] Machanavajjhala A, Kifer D, Gehrke J, Venkitasubramaniam M (2007). l-diversity: privacy beyond k-anonymity. ACM Trans. Knowl. Discov. Data (TKDD).

[115] Li, N., Li, T., Venkatasubramanian, S.: t-closeness: Privacy beyond k-anonymity and l-diversity,” Data Engineering, 2007. ICDE 2007. IEEE 23rd International Conference on, pp. 106–115 (2007)

[116] Aguilar Melchor, C., Gaborit, P.: A lattice based computationally efficient private information retrieval protocol. Cryptol ePrint Arch, Report, vol. 446 (2007)

[117] Chor, B., Gilboa, N.: Computationally private information retrieval. Proceedings of the twenty-ninth annual ACM symposium on Theory of computing, pp. 304–313 (1997)

[118] Goldberg, I.: Improving the robustness of private information retrieval. IEEE symposium on security and privacy, 2007. SP’07. pp. 131–148 (2007)

[119] Henry, R., Olumofin, F., Goldberg, I.: Practical pir for electronic commerce. *Proceedings of the 18th ACM conference on Computer and communications security*, pp. 677–690 (2011)

[120] Beimel A, Ishai Y, Malkin T (2004). Reducing the servers computation in private information retrieval: Pir with preprocessing. J. Cryptol..

[121] Gertner, Y., Goldwasser, S., Malkin, T.: A random server model for private information retrieval. Randomization and approximation techniques in computer science, Springer, pp. 200–217 (1998)

[122] Devet, C., Goldberg, I., Heninger, N.: Optimally robust private information retrieval. USENIX Security Symposium, pp. 269–283 (2012)

[123] Devet, C., Goldberg, I.: The best of both worlds: Combining information-theoretic and computational pir for communication efficiency. Privacy enhancing technologies (PETs), Springer, pp. 63–82, (2014)

[124] Fredrikson, M., Livshits, B.: Repriv: re-imagining content personalization and in-browser privacy. 2011 IEEE Symposium on Security and Privacy (SP), pp. 131–146 (2011)

[125] Guha, S., Cheng, B., Francis, P.: Privad: practical privacy in online advertising. 8th USENIX symposium on networked systems design and implementation (NSDI 11), (2011)

[126] Chen, R., Reznichenko, A., Francis, P., Gehrke, J.: Towards statistical queries over distributed private user data. Presented as part of the 9th USENIX symposium on networked systems design and implementation (NSDI 12), pp. 169–182 (2012)

[127] Chen, R., Akkus, I. E., Francis, P.: Splitx: high-performance private analytics. Proceedings of the ACM SIGCOMM 2013 conference on SIGCOMM, pp. 315–326 (2013)

[128] Tsang MM, Ho S-C, Liang T-P (2004). Consumer attitudes toward mobile advertising: an empirical study. Int. J. Electron. Commer..

[129] Merisavo M, Kajalo S, Karjaluoto H, Virtanen V, Salmenkivi S, Raulas M, Leppäniemi M (2007). An empirical study of the drivers of consumer acceptance of mobile advertising. J. Interact. Advert..

[130] Johnson GA, Shriver SK, Du S (2020). Consumer privacy choice in online advertising: who opts out and at what cost to industry?. Market. Sci..

[131] Dingledine, R., Mathewson, N., Syverson, P.: Tor: the second-generation onion router. Naval Research Lab, Washington DC (2004)

[132] Aggarwal, G., Bursztein, E., Jackson, C., Boneh, D.: An analysis of private browsing modes in modern browsers. USENIX Security Symposium, pp. 79–94 (2010)

[133] Akkus, I. E., Chen, R., Hardt, M., Francis, P., Gehrke, J.: Non-tracking web analytics. Proceedings of the 2012 ACM conference on computer and communications security (2012)

[134] Backes, M., Kate, A., Maffei, M., Pecina, K.: Obliviad: provably secure and practical online behavioral advertising. IEEE symposium on security and privacy (SP), pp. 257–271 (2012)

[135] Hardt, M., Nath, S.: Privacy-aware personalization for mobile advertising. Proceedings of the 2012 ACM conference on computer and communications security (2012)

[136] Samarati P, Sweeney L (1998). Generalizing data to provide anonymity when disclosing information. ACM Symp. Princ. Datab. Syst. (PODS).

[137] Pfitzmann A, Hansen M (2010). A terminology for talking about privacy by data minimization: anonymity, unlinkability, undetectability, unobservability, pseudonymity, and identity management.

[138] Ganta, S. R., Kasiviswanathan, S. P., Smith, A.: Composition attacks and auxiliary information in data privacy. Proceedings of the 14th ACM SIGKDD international conference on Knowledge discovery and data mining, pp. 265–273 (2008)

[139] Sweeney L (2000). Simple demographics often identify people uniquely. Health.

[140] Coull, S. E., Wright, C. V., Monrose, F., Collins, M. P., Reiter, M. K. * et al.*: Playing devil’s advocate: inferring sensitive information from anonymized network traces. Network and Distributed Systems Security (NDSS) Symposium, vol. 7, pp. 35–47 (2007)

[141] Artail H, Farhat R (2015). A privacy-preserving framework for managing mobile ad requests and billing information. IEEE Trans. Mob. Comput..

[142] Hardt, M., Nath, S.: Privacy-aware personalization for mobile advertising. Proceedings of the 2012 ACM conference on Computer and communications security, pp. 662–673 (2012)

[143] Wermke, D., Huaman, N., Acar, Y., Reaves, B., Traynor, P., Fahl, S.: A large scale investigation of obfuscation use in google play. Proceedings of the 34th annual computer security applications conference, pp. 222–235 (2018)

[144] Weinsberg, U., Bhagat, S., Ioannidis, S., Taft, N.: Blurme: inferring and obfuscating user gender based on ratings. Proceedings of the sixth ACM conference on Recommender systems, pp. 195–202 (2012)

[145] Salamatian, S., Zhang, A., du Pin Calmon, F., Bhamidipati, S., Fawaz, N., Kveton, B., Oliveira, P., Taft, N.: How to hide the elephant-or the donkey-in the room: Practical privacy against statistical inference for large data. IEEE Global Conference on Signal and Information Processing (GlobalSIP) (2013)

[146] du Pin Calmon, F., Fawaz, N.: Privacy against statistical inference. 50th Annual Allerton Conference on IEEE communication, control, and computing (Allerton), pp. 1401–1408 (2012)

[147] Li, C., Shirani-Mehr, H., Yang, X.: Protecting individual information against inference attacks in data publishing. Advances in databases: concepts, systems and applications, Springer, pp. 422–433 (2007)

[148] Howe, D.C., Nissenbaum, H.: Trackmenot: resisting surveillance in web search. Lessons from the identity trail: anonymity, privacy, and identity in a networked society, pp. 417–436 (2009)

[149] Agrawal R, Srikant R (2000). Privacy-preserving data mining. ACM Sigmod Rec..

[150] Evfimievski, A., Gehrke, J., Srikant, R.: Limiting privacy breaches in privacy preserving data mining. Proceedings of the twenty-second ACM SIGMOD-SIGACT-SIGART symposium on Principles of database systems, pp. 211–222 (2003)

[151] Kargupta, H., Datta, S., Wang, Q., Sivakumar, K.: On the privacy preserving properties of random data perturbation techniques. Third IEEE International Conference on Data Mining, 2003. ICDM 2003. pp. 99–106 (2003)

[152] Mor, N., Riva, O., Nath, S., Kubiatowicz, J.: Bloom cookies: web search personalization without user tracking. Network and Distributed Systems Security (NDSS) Symposium, (2015)

[153] Bloom BH (1970). Space/time trade-offs in hash coding with allowable errors. Commun. ACM.

[154] Dwork, C., McSherry, F., Nissim, K., Smith, A.: Calibrating noise to sensitivity in private data analysis. Theory of cryptography conference, Springer, pp. 265–284 (2006)

[155] Dwork C, Roth A (2014). The algorithmic foundations of differential privacy. Found. Trends Theor. Comput. Sci..

[156] Cho, H., Ippolito, D., Yu, Y. W.: Contact tracing mobile apps for covid-19: Privacy considerations and related trade-offs. Europe PMC (2020)

[157] Yan Y, Gao X, Mahmood A, Feng T, Xie P (2020). Differential private spatial decomposition and location publishing based on unbalanced quadtree partition algorithm. IEEE Access.

[158] Zhang, X., Chen, R., Xu, J., Meng, X., Xie, Y.: Towards accurate histogram publication under differential privacy. Proceedings of the 2014 SIAM international conference on data mining, pp. 587–595 (2014)

[159] Zhang, J., Xiao, X., Xie, X.: Privtree: A differentially private algorithm for hierarchical decompositions. Proceedings of the 2016 International Conference on Management of Data, pp. 155–170 (2016)

[160] Dwork, C.: Differential privacy, Automata, languages and programming, Springer, pp. 1–12, (2006)

[161] Dierks, T., Rescorla, E.: The transport layer security (TLS) protocol version 1.2. https://www.rfc-editor.org/rfc/rfc5246 (2008)

[162] Rastogi, V., Nath, S.: Differentially private aggregation of distributed time-series with transformation and encryption. Proceedings of the 2010 ACM SIGMOD international conference on management of data, pp. 735–746 (2010)

[163] Shi, E., Chan, T. H., Rieffel, E., Chow, R., Song, D.: Privacy-preserving aggregation of time-series data. Proceedings network and distributed systems security (NDSS) symposium, vol. 2, pp. 1–17 (2011)

[164] Kushilevitz, E., Ostrovsky, R.: Replication is not needed: Single database, computationally-private information retrieval. IEEE Computer Society, IEEE 54th annual symposium on foundations of computer science, p. 364 (1997)

[165] Chor, B., Goldreich, O., Kushilevitz, E., Sudan, M.: Private information retrieval. IEEE computer society, proceedings of the 36th annual symposium on foundations of computer science, p. 41, (1995)

[166] Chor, B., Gilboa, N., Naor, M.: Private information retrieval by keywords. Citeseer https://citeseerx.ist.psu.edu/document?repid=rep1 &type=pdf &doi=70d2a37d5af527dfc345691e2f978f6e46dc4efe (1997)

[167] Chaum DL (1981). Untraceable electronic mail, return addresses, and digital pseudonyms. Commun. ACM.

[168] Desmedt, Y., Kurosawa, K.: How to break a practical mix and design a new one. International conference on the theory and applications of cryptographic techniques, Springer, pp. 557–572 (2000)

[169] Chu C-K, Tzeng W-G (2008). Efficient k-out-of-n oblivious transfer schemes. J. Univers. Comput. Sci..

[170] Naor, M., Pinkas, B.: Oblivious transfer and polynomial evaluation. Proceedings of the thirty-first annual ACM symposium on Theory of computing, pp. 245–254 (1999)

[171] Boudot, F.: Efficient proofs that a committed number lies in an interval. Advances in Cryptology-EUROCRYPT 2000, international conference on the theory and applications of cryptographic techniques, Springer, pp. 431–444, (2000)

[172] Schnorr, C.-P.: Efficient identification and signatures for smart cards. Advances in cryptology-CRYPTO’89 proceedings, Springer, pp. 239–252 (1990)

[173] Brands SA (2000). Rethinking public key infrastructures and digital certificates: building in privacy.

[174] Camenisch, J., Michels, M.: Proving in zero-knowledge that a number is the product of two safe primes. Advances in Cryptology-EUROCRYPT’99, international conference on the theory and applications of cryptographic techniques, Springer, pp. 107–122 (1999)

[175] Ghaderi, J., Srikant, R.: Towards a theory of anonymous networking. INFOCOM, 2010 Proceedings IEEE, pp. 1–9 (2010)

[176] Abe, M.: Universally verifiable mix-net with verification work independent of the number of mix-servers. International Conference on the Theory and Applications of Cryptographic Techniques, Springer, pp. 437–447 (1998)

[177] Piotrowska, A. M.: Low-latency mix networks for anonymous communication. PhD thesis, UCL (University College London), (2020)

[178] Abe, M.: Mix-networks on permutation networks. International conference on the theory and application of cryptology and information security, Springer, pp. 258–273 (1999)

[179] Jakobsson, M.: A practical mix. International conference on the theory and applications of cryptographic tecniques, Springer, pp. 448–461, (1998)

[180] Jakobsson, M., Juels, A.: Millimix: mixing in small batches. Center for discrete mathematics and theoretical computer science (DIMACS), Technical report 99-33, https://www.arijuels.com/wp-content/uploads/2013/09/JJ99b.pdf (1999)

[181] Mitomo, M., Kurosawa, K.: Attack for flash mix. International conference on the theory and application of cryptology and information security, pp. 192–204 (2000)

[182] Goldreich, O., Micali, S., Wigderson, A.: How to play any mental game. Proceedings of the nineteenth annual ACM symposium on Theory of computing, pp. 218–229 (1987)

[183] Juels, A.: Targeted advertising... and privacy too. Topics in Cryptology CT-RSA, Springer, **2001**, pp. 408–424 (2001)

[184] Yi X, Paulet R, Bertino E (2014). Homomorphic encryption and applications.

[185] Erkin Z, Veugen T, Toft T, Lagendijk RL (2012). Generating private recommendations efficiently using homomorphic encryption and data packing. IEEE Trans. Inf. Forensics Secur.

[186] Badsha S, Yi X, Khalil I (2016). A practical privacy-preserving recommender system. Data Sci. Eng..

[187] Badsha, S., Yi, X., Khalil, I., Bertino, E.: Privacy preserving user-based recommender system. 2017 IEEE 37th international conference on Distributed Computing Systems (ICDCS), pp. 1074–1083 (2017)

[188] Cramer, R., Damgård, I.: Multiparty computation, an introduction. Contemporary cryptology, Springer, pp. 41–87 (2005)

[189] P. E. project D5.2, Papaya: platform for privacy preserving data analytics. https://www.papaya-project.eu/node/163, (2022)

[190] Canard, S., Desmoulins, N., Hallay, S., Hamdi, A., Le Hello, D.: Westat: a privacy-preserving mobile data usage statistics system. Proceedings of the 2021 ACM Workshop on Security and Privacy Analytics, pp. 5–14 (2021)

[191] Kosba, A., Miller, A., Shi, E., Wen, Z., Papamanthou, C.: Hawk: the blockchain model of cryptography and privacy-preserving smart contracts. 2016 IEEE symposium on security and privacy (SP), pp. 839–858 (2016)

[192] Wood, G., et al.: Ethereum: a secure decentralised generalised transaction ledger. Ethereum project yellow paper **151**(2014), pp. 1–32 (2014)

[193] Vukolić, M.: The quest for scalable blockchain fabric: Proof-of-work vs. bft replication. International workshop on open problems in network security, Springer, pp. 112–125, (2015)

[194] Dorri A, Steger M, Kanhere SS, Jurdak R (2017). Blockchain: a distributed solution to automotive security and privacy. IEEE Commun. Mag..

[195] Nakamoto, S.: Bitcoin: a peer-to-peer electronic cash system. Technical Report, Manubot, (2019)

[196] Dedeoglu, V., Jurdak, R., Dorri, A., Lunardi, R., Michelin, R., Zorzo, A., Kanhere, S.: Blockchain technologies for iot. Advanced Applications of Blockchain Technology, Springer, pp. 55–89, (2020)

[197] Tandon A, Dhir A, Islam N, Mäntymäki M (2020). Blockchain in healthcare: a systematic literature review, synthesizing framework and future research agenda. Comput. Ind..

[198] Chen Y, Bellavitis C (2020). Blockchain disruption and decentralized finance: the rise of decentralized business models. J. Bus. Ventur. Insights.

[199] Yang J, Wen J, Jiang B, Wang H (2020). Blockchain-based sharing and tamper-proof framework of big data networking. IEEE Netw..

[200] Freudiger, J., Vratonjic, N., Hubaux, J.-P.: “Towards privacy-friendly online advertising,” IEEE Web 2.0 Security and Privacy (W2SP), no. LCA-CONF-2009-008, (2009)

[201] Akkus, I. E., Chen, R., Hardt, M., Francis, P., Gehrke, J.: Non-tracking web analytics. Proceedings of the 2012 ACM conference on computer and communications security, pp. 687–698, (2012)

[202] Christopher, S., Sid, S., Dan, K.: Do Not Track (DNT). https://donottrack-doc.com/en/intro/ (2022)

[203] Ghosh A, Roth A (2013). Selling privacy at auction. Games Econ. Behav..

[204] Riederer, C., Erramilli, V., Chaintreau, A., Krishnamurthy, B., Rodriguez, P.: For sale: your data: by: you. Proceedings of the 10th ACM workshop on hot topics in networks, p. 13 (2011)

[205] Bashir, M. A., Arshad, S., Robertson, W., Wilson, C.: Tracing information flows between ad exchanges using retargeted ads. 25th USENIX Security Symposium (USENIX Security 16), pp. 481–496 (2016)

[206] Melicher W, Sharif M, Tan J, Bauer L, Christodorescu M, Leon PG (2016). (Do Not) Track me sometimes: users’ contextual preferences for Web tracking. Proc. Priv. Enhanc. Technol. (PETs).

[207] Mozaffari, H., Houmansadr, A.: Heterogeneous private information retrieval. Network and Distributed Systems Security (NDSS) Symposium (2020)

[208] Shamir A (1979). How to share a secret. Commun. ACM.

[209] Guruswami V, Rudra A (2008). Explicit codes achieving list decoding capacity: error-correction with optimal redundancy. IEEE Trans. Inf. Theory.

[210] Mittal, P., Olumofin, F. G., Troncoso, C., Borisov, N., Goldberg, I.: Pir-tor: scalable anonymous communication using private information retrieval. USENIX Security Symposium (2011)

[211] Beimel, A., Stahl, Y.: Robust information-theoretic private information retrieval. Security in Communication Networks, Springer, pp. 326–341, (2003)

[212] Beimel A, Stahl Y (2007). Robust information-theoretic private information retrieval. J. Cryptol..

[213] Micali, S., Peikert, C., Sudan, M., Wilson, D. A.: Optimal error correction against computationally bounded noise, Theory of Cryptography, pp. 1–16, (2005)

[214] Boneh, D., Lynn, B., Shacham, H.: Short signatures from the weil pairing. Advances in Cryptology-ASIACRYPT 2001, international conference on the theory and application of cryptology and information security, Springer, pp. 514–532 (2001)

[215] Kate, A., Zaverucha, G. M., Goldberg, I.: Constant-size commitments to polynomials and their applications. Advances in cryptology-ASIACRYPT 2010, international conference on the theory and application of cryptology and information security, Springer, pp. 177–194, (2010)

[216] Kate, A., Zaverucha, G. M., Goldberg, I.: Polynomial commitments. Technical report centre for applied cryptographic research (CACR) 2010-10, University of Waterloo, https://cacr.uwaterloo.ca/techreports/2010/cacr2010-10.pdf, (2010)

[217] Bellare, M., Garay, J. A., Rabin, T.: Fast batch verification for modular exponentiation and digital signatures. Advances in cryptology-EUROCRYPT’98, international conference on the theory and applications of cryptographic techniques, Springer, pp. 236–250 (1998)

[218] Bellare, M., Garay, J. A., Rabin, T.: Batch verification with applications to cryptography and checking. Springer, LATIN’98: Theoretical Informatics, pp. 170–191 (1998)

[219] Samarati, P., Sweeney, L.: Protecting privacy when disclosing information: k-anonymity and its enforcement through generalization and suppression,” Technical Report, SRI International, https://dataprivacylab.org/dataprivacy/projects/kanonymity/paper3.pdf (1998)

